# Deep learning methods for 2D material electronic properties

**DOI:** 10.1039/d5dd00155b

**Published:** 2025-12-09

**Authors:** Artem Mishchenko, Anupam Bhattacharya, Xiangwen Wang, Henry Kelbrick Pentz, Yihao Wei, Qian Yang

**Affiliations:** a Department of Physics and Astronomy, University of Manchester Manchester UK artem.mishchenko@manchester.ac.uk anupam.bhattacharya@manchester.ac.uk

## Abstract

This review explores the impact of deep learning (DL) techniques on understanding and predicting electronic structures in two-dimensional (2D) materials. We highlight unique computational challenges posed by 2D materials and discuss how DL approaches – such as physics-aware models, generative AI, and inverse design – have significantly improved predictions of critical electronic properties, including band structures, density of states, and quantum transport phenomena. Through selected case studies, we illustrate how DL methods accelerate discoveries in emergent quantum phenomena, topology, superconductivity, and autonomous materials exploration. Finally, we outline promising future directions, stressing the need for robust data standardization and advocating for integrated frameworks that combine theoretical modeling, DL methods, and experimental validations.

## Abstract 2

This review explores the impact of deep learning (DL) techniques on understanding and predicting electronic structures in two-dimensional (2D) materials.

## Graphical abstract

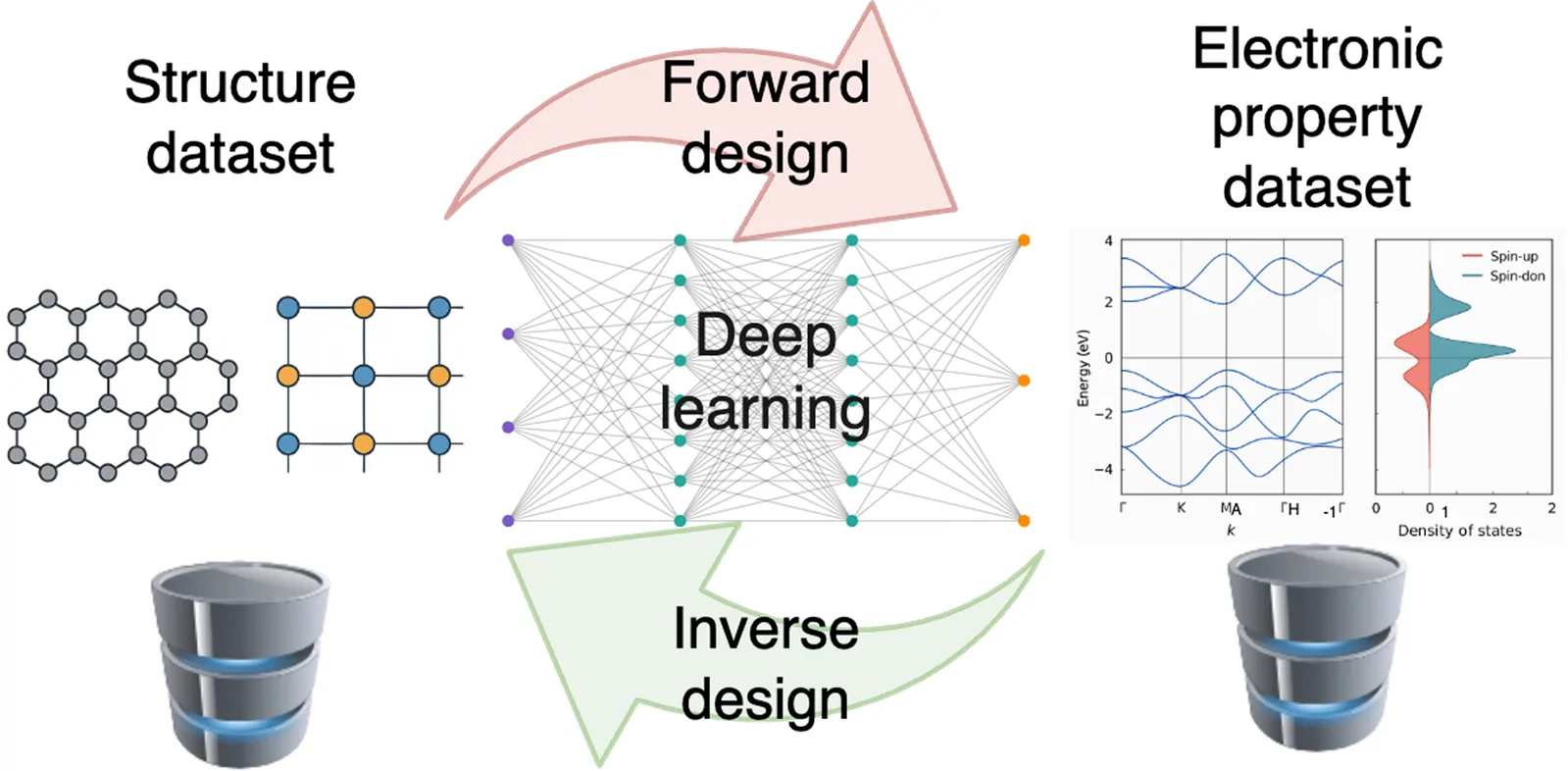

## 1 Introduction

Two-dimensional (2D) materials offer diverse applications in (opto)electronics, energy storage, catalysis, sensing, and quantum technologies. Their reduced dimensionality leads to properties such as tunable electronic band structures, strong light–matter interactions, and high catalytic activity.^1–3^ For 2D materials to continue to drive innovation, accurate and efficient predictions of their electronic structures are paramount for both fundamental understanding and technological applications.

Despite their conceptual simplicity compared to bulk materials, modeling the electronic structure of 2D systems presents unique challenges. Most computational approaches rely on 3D periodic boundary conditions, requiring a large vacuum along the out-of-plane direction to prevent artificial interactions between repeated layers. However, there is no widely established method designed specifically for 2D systems – that is, periodic within the plane but non-periodic out-of-plane. This limitation affects both accuracy and computational costs, particularly for multilayer 2D (hetero)structures, moiré superlattices, and strongly correlated systems.^4^ The growing shift toward automated, closed-loop materials discovery laboratories further underscores the need for computationally efficient methods, as rapid screening is critical for accelerating breakthroughs.^5–7^

Traditional electronic structure methods, such as tight-binding models and density functional theory (DFT), have long been used to study 2D materials. Tight binding models are computationally efficient and offer some analytical insights but fail to capture many-body interactions and other complex effects. DFT, while more robust, struggles with accurately modeling van der Waals (vdW) interactions – which are crucial in layered heterostructures^8,9^ – and remains computationally expensive, limiting its feasibility for large-scale high-throughput studies.

To overcome these limitations, artificial intelligence (AI), particularly deep learning (DL), has emerged as a powerful tool for electronic structure prediction. Unlike traditional methods, which rely on explicitly defined physical models, DL learns structure–property relationships directly from data, enabling it to approximate computationally demanding calculations at a fraction of the cost. A recent comprehensive perspective further emphasizes strategies for AI-driven research in chemistry and materials science.^10^ Indeed, recent studies have shown that DL models can improve predictive accuracy by learning from diverse datasets, including both high-fidelity simulations and experimental measurements.^11–13^

This review explores how DL is transforming electronic structure modeling in 2D materials, addressing key computational challenges and accelerating materials discovery. We begin with an overview of traditional computational and experimental approaches, followed by a discussion of databases and data representations used in training AI models. We then examine DL-driven predictions of electronic properties, such as band structures, density of states, and quantum transport phenomena, distinguishing between forward design, which predicts material properties from known structures, and inverse design, which identifies materials with target functionalities. We further differentiate between strictly inverse design (non-generative), which selects materials from existing databases, and generative inverse design, where AI models propose entirely novel structures using methods such as variational autoencoders (VAEs) and generative adversarial networks (GANs).

Beyond property prediction, we discuss how DL aids in discovering emergent quantum phenomena, including nontrivial topology, strongly correlated phases, and moiré superlattices. We also highlight the challenges in integrating DL with first-principles methods and experimental validation, emphasizing the need for improved interpretability, generalization, and data standardization. Finally, we outline future directions, including foundation models, AI-driven automation in experiments, and the integration of DL with quantum computing, providing a comprehensive perspective on how DL is shaping the future of 2D materials research.

We emphasize that this is a rapidly evolving field, with a continual influx of new publications. Consequently, it is not feasible to provide an exhaustive account of all developments. Instead, we highlight a selection of recent review articles that cover adjacent areas and complement the scope of this work. We recommend the review by Malica *et al.* for details of AI-assisted synthesis and interpretability of experiments of materials,^14^ Vital *et al.* for details of machine learning (ML) based interatomic potentials,^15^ the overview of numerical techniques for layered materials by Gray and Herbert,^16^ and new research avenues in electronic structure analysis with AI summarized by Kulik *et al.*^17^

## 2 Foundations for DL in 2D materials

### 2.1 Electronic band structures of 2D materials: key concepts and computational challenges

Electrons in materials are most commonly represented as wavefunctions that satisfy Schrödinger's equation, which can be solved to yield eigenvalues *E* and eigenvectors or wave functions *Ψ*. For materials with many electrons, solving this equation is practically impossible due to the complex nature of many-body electronic interactions. Various numerical techniques have been developed to address this challenge, including Hartree–Fock (HF), Density Functional Theory (DFT), Dynamic Mean-Field Theory (DMFT), Coupled Cluster (CC) theory, and Quantum Monte-Carlo (QMC) methods.^18^ DFT, the workhorse of computational materials science, handles many-body physics by mapping the complex many-electron problem onto a simpler system of non-interacting electrons moving in an effective potential, with electron–electron interactions approximated through exchange-correlation potentials that capture quantum mechanical effects.

While these computational methods were originally developed for bulk 3D materials, they can be adapted to 2D materials by restricting periodic boundary conditions (PBC) to two dimensions. However, for multilayer 2D materials, accurately modeling van der Waals interactions between layers remains an active area of research.^16^

The electronic band structure of a material is typically represented through its dispersion relation – the relationship between energy eigenvalues and wave vectors. Conventionally, these energies are plotted as a function of wave vectors *k* (or momenta *p* = *ℏk*, where *ℏ* is the reduced Planck's constant) along high-symmetry lines in the reciprocal space, forming what is called the electronic band structure. For 2D materials, these high-symmetry lines lie in a plane, reflecting their reduced dimensionality.

Materials databases store this electronic structure information in various formats. Most commonly, dispersion relations along high-symmetry paths are stored as electronic band structures. Some databases also provide electronic dispersion on uniform k-grids, as a result, requiring significantly more storage space. Another common representation is the Density of States (DOS), which quantifies the occupation of electronic states at different energies and momenta, and is often presented alongside band structures. More detailed representations include electronic wave functions and charge density distributions (*Ψ*^2^), which would undoubtedly require substantially more storage capacity.

Complementing computational approaches are various experimental techniques that can directly probe the electronic structure of 2D materials. Spectroscopic methods such as X-ray Photoemission Spectroscopy (XPS), X-ray Absorption Spectroscopy (XAS), Ultraviolet Photoelectron spectroscopy (UPS), and Ultraviolet-visible-Near-Infrared spectroscopy (UV-Vis/NIR) provide valuable information about energies of electronic states in 2D materials.^19^ Techniques like Electron Energy Loss Spectroscopy (EELS)^20^ and Low Energy Electron Diffraction (LEED)^21^ have also proven instrumental in identifying electronic structures of 2D materials. Methods like Scanning Tunneling Microscopy (STM) allow direct visualization of local DOS over 2D surfaces with atomic resolution. Momentum-resolved techniques, particularly Angle-Resolved Photoemission Spectroscopy (ARPES) and Quasi-Particle Interference (QPI), offer unique capabilities to simultaneously measure electron energies and momenta, providing comprehensive mapping of band structures in reciprocal space.

So far, a wide range of computational and experimental methods are available to determine electronic structures of 2D materials, producing vast data stored in various formats across different platforms. In general, current computational datasets provide more structured information that could be systematically categorized. However, to predict properties of new materials from existing knowledge, AI approaches are required, as the volume of data and the complex interdependencies between variables far exceed the capabilities of conventional analytical methods.

The challenge intensifies when it comes to experimental data, which are largely unstructured and dispersed throughout scientific literature. AI techniques become even more critical for effective information extraction, data curation, standardization across sources, and accurate interpolation and extrapolation for prediction purposes. In the following subsections, we examine the existing databases, data representation formats, and computational tools for mining this wealth of electronic structure information.

### 2.2 Databases and data curation

The advancement of first-principles calculations has enabled the development of comprehensive computational databases, which have become indispensable tools for electronic structure exploration.^22^ These databases provide large-scale, standardized datasets that accelerate both materials discovery and machine learning model development. While major repositories such as the Materials Project^23^ and JARVIS-DFT^24^ were initially designed for 3D materials, they contain valuable information on 2D systems as well. Researchers can extract band structures and DOS, specific to monolayers or layered structures, by filtering database entries for reduced-dimensionality systems. This versatility extends the utility of these general-purpose databases to 2D materials research, yet challenges still exist in accurately identifying and integrating 2D-specific data, particularly when materials exhibit properties that differ between their bulk and 2D forms.

To address the need for specialized 2D materials information, dedicated databases have emerged as focal points for the research community. The Computational 2D Materials Database (C2DB),^25,26^ Materials Cloud 2D Database (MC2D),^27,28^ and 2D Materials Encyclopedia (2DmatPedia)^29^ provide comprehensive catalogs of crystal structures, phonon dispersions, electronic band structures, and optical properties. These representations contain thousands of 2D materials with properties predicted using DFT and many-body perturbation theory methods.

Expanding beyond monolayers, computational studies have demonstrated the rich potential of stacking engineering in 2D materials.^30,31^ For instance, over 2500 (out of 8000) stable homo-bilayers structures with emergent properties distinct from their monolayer constituents are identified through high-throughput DFT,^30^ highlighting the extent which stacking engineering leads to novel physics and functionalities. Many homo-bilayers exhibit multiple stable stacking configurations, giving rise to sliding ferroelectricity – a phenomenon where the relative displacement of layers breaks the inversion symmetry and induces out-of-plane polarization. This effect has been demonstrated in various systems, including bilayer boron nitride, rhombohedral-stacked transition metal chalcogenides, and marginally twisted 2D materials.^32^ Recent developments in DFT-based high-throughput studies have extended this further to hetero-bilayers,^33,34^ offering insight into interfacial band alignment in van der Waals heterostructures. Computational materials databases, including those for 2D materials, are summarized in Table 1.

**Table 1:** Popular open-access databases of materials. A more detailed list of 3D materials databases may be found at https://github.com/sedaoturak/data-resources-for-materials-science.git

	Database	Description	# Of entries	URL
3D	Materials Project^23^	A database of computed materials properties for research	170 000+	https://www.materialsproject.org
	Automatic FLOW for materials discovery (AFLOW)	Largest 3D materials database based on VASP calculations	3.5 million	https://www.aflowlib.org
	JARVIS-DFT^24^	A repository of VASP-calculated properties for mostly 3D materials and 1000 2D materials	80 000+	https://jarvis.nist.gov
	Crystallography open database (COD)	Database of experimentally observed crystal structures	500 000+	https://www.crystallography.net/cod/
	Open quantum materials database (OQMD)^35^	VASP calculated material properties database	300 000+	https://www.oqmd.org
	Cambridge structural database (CSD)	Large experimental structure database including XRD, neutron diffraction	1.25 million	https://www.ccdc.cam.ac.uk/structures/
	GNoME dataset^36^	AI predicted mosty 3D and some 2D stable materials	2.2 million	https://github.com/google-deepmind/materials_discovery.git
2D	Computational 2D materials database (C2DB)^25,26^	A database of properties for 2D materials calculated using DFT code GPAW	4000: DFT 11600: AI	https://www.cmr.fysik.dtu.dk/c2db/c2db.html
	Materials cloud 2D crystals database (MC2D)^27,28^	2D crystal structures exfoliated from 3D; DFT simulation with QE	3077	https://mc2d.materialscloud.org
	2D materials encyclopedia (2DMatPedia)^29^	2D materials created with top–down and bottom–up approaches from materials project; DFT calculations with VASP	6351	https://www.2dmatpedia.org
	Virtual 2D materials database (V2DB)^37^	A database of AI generated likely-stable 2D materials with key properties	316 505: AI	https://doi.org/10.7910/DVN/SNCZF4
	MatHub-2D^38^	VASP and phonopy calculation results on high mobility semiconductors	1900	http://www.mathub2d.net/materials/matdb
	2D octahereal materials database (aNANt)^39^	Functional 2D materials simulated using VASP	3099	https://anant.mrc.iisc.ac.in/apps/2D
	Topological 2D materials database (2D-TQCDB)^40^	DFT with VASP (with SOC) hosting band structures and detail topological properties	8872	https://topologicalquantumchemistry.com/topo2d/index.html
	Ferromagnetic 2D materials^41^	VASP calculation identifying nonmagnetic to ferromagnetic transition *via* hole doping	122	SI of ref. 41
	2D topological insulators^42^	DFT calculations with QE (with SOC) materials from MC2D	1825	https://www.materialscloud.org/discover/2dtopo/dashboard/ptable
	Experimental 2D materials^43^	List of reported experimentally synthesized 2D materials	300+	https://zenodo.org/records/10887700
	2D materials platform^44^	Experimental results such as XPS, RHEED, Raman, AFM on 2D materials	—	https://2dmat.chemdx.org/
Homo-bilayer	van der Waals bilayer database (BiDB)^30^	A database of van der Waals bilayer material properties calculated with GPAW	2586	https://cmr.fysik.dtu.dk/bidb/bidb.html
	Bilayer materials DataSet (BMDS)^31^	Band structures calculated with SOC using VASP	760	BMDS
Hetero-bilayers	InterMatch^45^	Calculates interface properties like mismatch, charge transfer from bulk properties	—	https://contribs.materialsproject.org/projects/intermatch/
	Layered heterostructures on materials project^46^	Active learning platform to compute electronic property of heterostructures	—	https://magics.usc.edu/data/

Despite the growing availability of databases for 2D materials, consolidating data for machine learning applications remains challenging. Researchers frequently need to extract and integrate data from multiple databases to assemble comprehensive training datasets. However, issues such as data duplication and inconsistencies intensify as these repositories rapidly expand. Different DFT functionals used across databases result in slight but evident variations in the predicted crystal structures and properties for nominally identical materials. Moreover, disparate representations of physical properties – such as differing conventions for elastic tensors – further compromise data compatibility and comparability. Without robust deduplication and harmonization strategies, repeated occurrences of similar or identical materials with varying properties may inadvertently introduce biases into machine learning models.

A notable example of addressing these challenges comes from Meng *et al.*,^41^ who developed a systematic approach to data integration. The authors collected 2D crystal structures from 2DmatPedia, C2DB, and Materials Cloud to identify non-magnetic 2D semiconductors with potential for hole-induced ferromagnetism. Their high-throughput screening process incorporated critical data cleaning steps: exclusion of magnetic metals, identification and removal of duplicates across databases, and filtering out materials with low thermodynamic stability. This methodical approach yielded a curated dataset of 3000 materials for further study, demonstrating a proper data cleaning workflow that strengthens materials discovery.

While computational databases provide structured insights, experimental data from the published literature constitute a critical complementary resource that offers essential real-world validation. Techniques such as ARPES, STM, and XPS could generate rich, realistic datasets capturing the true behavior of materials beyond idealized simulations. However, extracting and curating high-quality experimental data presents even greater challenges, including variability in experimental setups, inconsistencies in sample quality, non-uniform reporting standards, and the substantial costs and time required for such experiments.^47^ Addressing these challenges requires robust pre-processing pipelines and standardized data formats to assess measurement uncertainties, filtering out experimental noise, and assembling high-confidence datasets suitable for machine learning applications. The development of community-wide data standardization protocols would significantly accelerate the integration of experimental and computational data, representing a frontier opportunity to develop more robust and transferable predictive models.

### 2.3 Data representation

Data representation refers to the process of encoding the structural, electronic, or other properties of materials into mathematical forms that can be understood and processed by ML algorithms. Effective representation of data is central to the success of DL models in understanding and predicting the electronic structures of 2D materials. The choice of representation determines what information the model can learn, its ability to generalize, and the physical interpretability of its predictions. In this section, we discuss the key material representations used in DL workflows for 2D materials, categorized into structural, electronic, and hybrid representations.

#### 2.3.1 Crystal structure representations

Structural representations encode the atomic configuration and the crystal structure of materials, which fundamentally determine their electronic and physical properties. For 2D materials, these representations must efficiently capture both in-plane interactions and the reduced dimensionality.

**Fig. 1:**
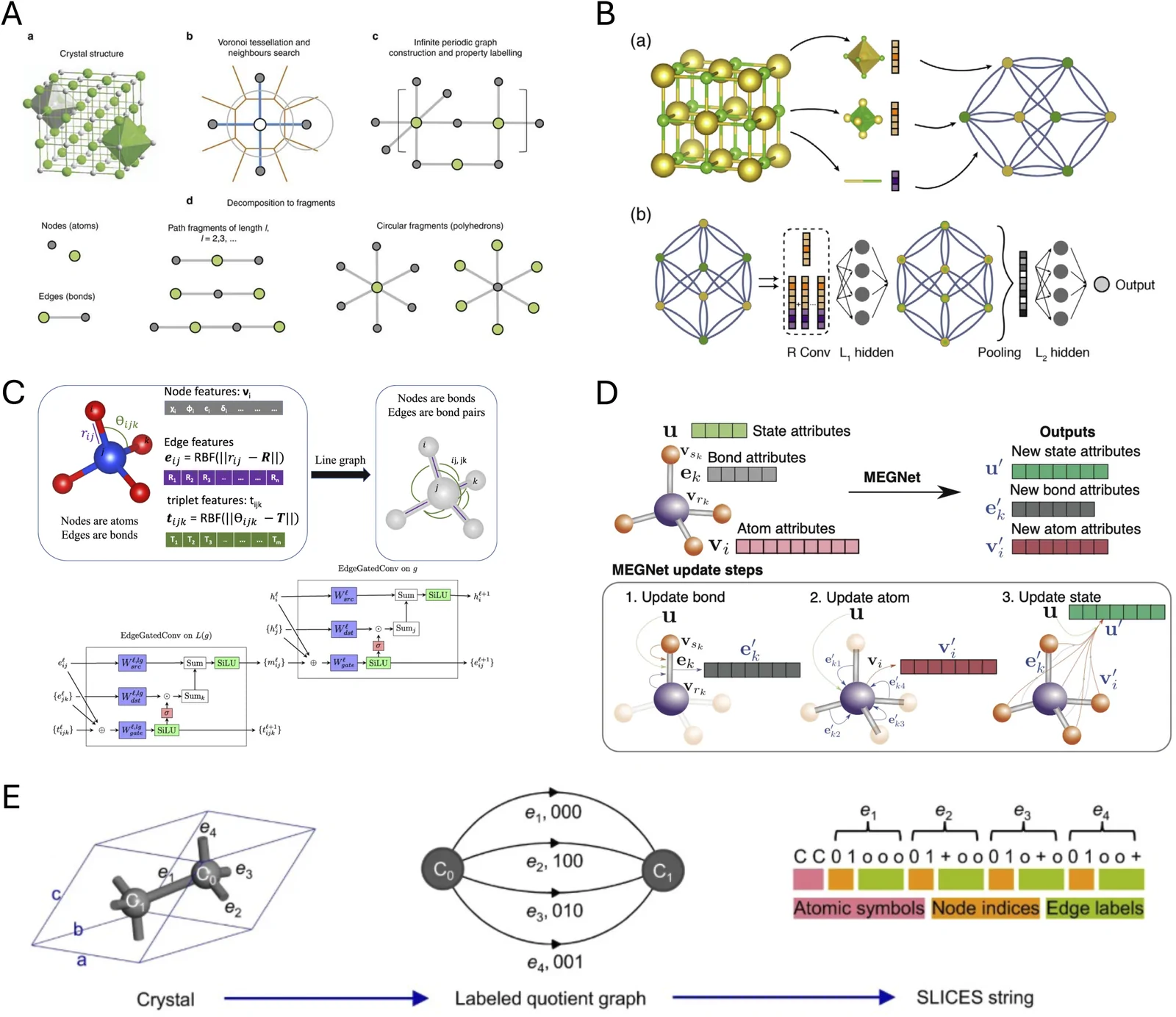
Crystal structure representations. (A) Property-Labeled Materials Fragments (PLMFs), adapted from ref. 48 with permission from Springer Nature, *Nat. Commun.*, 2017. (B) Crystal Graph Convolutional Neural Network (CGCNN), adapted from ref. 49 with permission from American Physical Society, *Phys. Rev. Lett.*, 2018. (C) Atomistic Line Graph Neural Network (ALIGNN), reproduced from ref. 50 with permission from Springer Nature, *npj Comput. Mater.*, 2021. (D) Materials Graph Network (MEGNet), adapted from ref. 51 with permission from American Chemical Society, *Chem. Mater.*, 2019. (E) Simplified Line-Input Crystal-Encoding System (SLICES), adapted from ref. 52 with permission from Springer Nature, *Nat. Commun.*, 2023.

Graph-based representations have emerged as the predominant approach for encoding atomic structures, where atoms are represented as nodes and their interactions as edges. Isayev *et al.*^48^ pioneered this approach with property-labeled materials fragments (PLMFs), which use Voronoi tessellation and covalent radii cutoffs to partition crystal structures into meaningful subunits labeled with elemental properties such as valence electron count and electronegativity, creating a structural representation for predicting electronic properties, Fig. 1A. Almost simultaneously, neural message passing pioneered by Gilmer *et al.*,^53^ provided a natural, powerful, and flexible way to capture both local chemical environments and long-range atomic interactions by updating NNs through sending nodal information only along graph edges. This was one of the first use of graph neural networks (GNNs) in electronic structure prediction. Other early GNNs for materials, such as the crystal graph convolutional neural network (CGCNN),^49^ build a convolutional architecture on top of crystal graphs where atoms are nodes and bonds are edges, using iterative message passing to capture local chemical environments and predict material properties with DFT-level accuracy, including formation energy, band gap, or elastic modulus, Fig. 1B.

Building upon this foundation, more sophisticated graph architectures have been developed. The atomistic line graph neural network (ALIGNN)^50^ extends CGCNN by performing message passing on both the interatomic bond graph and its line graph corresponding to bond angles, explicitly incorporating angular information to improve prediction accuracy for diverse materials properties, Fig. 1C. MatErials Graph Network (MEGNet)^51^ further enriches graph representations by including global state attributes such as temperature, pressure, or entropy, alongside atom and bond features, allowing for more accurate prediction of materials properties at various thermodynamic conditions, Fig. 1D. AtomSets^54^ goes further by treating atoms and bonds as unordered sets rather than fixed graph elements, providing greater flexibility and making it better suited for diverse material representations without atomic ordering constraints. MatterGen, introduced by Zeni *et al.*,^55^ presents a diffusion-based generative model that encodes materials universally as a combination of atomic types, lattice vectors, and fractional atomic coordinates within the unit cell. This representation ensures invariance to permutations, translations, rotations, and supercell transformations, while achieving remarkable performance in generating stable, unique, and new inorganic materials across the periodic table with properties closely matching DFT predictions.

While graph-based representations are more intuitive for crystal structures, string-based representation allows researchers to take advantage of the extensive and rapidly evolving field of natural language processing. The Simplified Line-Input Crystal-Encoding System (SLICES),^52^ shown in Fig. 1E, is a string-based crystal representation analogous to SMILES for molecules,^56^ offering both invertibility and invariance to transformations. SLICES encodes compositional and topological data of crystal structures, successfully reconstructing 94.95% of over 40 000 diverse crystal structures. This representation facilitates proof-of-concept inverse design studies in solid-state materials, for example, exploring candidates for direct narrow-gap semiconductors in optoelectronics, while the quantitative accuracy of such applications ultimately depends on the reliability of the underlying forward bandgap predictors.

Very recently, sequence models have also started using Wyckoff representation as input strings. Wyckoff representation of atomic coordinates is easy to put into a sequence for easy integration with a transformer. It also encodes the symmetries of the crystallographic sites catering to providing physical insight to the model.^57–59^

Topology-based methods, such as those described in Chen *et al.*^60^ and Jiang *et al.*,^61^ utilize persistent homology to encode atomic configurations and their interactions into simplified topological descriptors. These representations effectively capture both the in-plane structural relationships and reduced dimensionality, complementing graph-based approaches by providing a powerful framework for predicting electronic and physical properties with enhanced accuracy.

Physical property-based representations focus on encoding key electronic, vibrational, and optical properties of 2D materials. The Coulomb matrix is a widely used descriptor that encodes atomic interactions as the Coulomb potential between nuclei. However, its effectiveness is limited by its sensitivity to atomic ordering, leading to the development of several improved variants.^62,63^ Key approaches include the randomly sorted Coulomb matrix, which generates multiple permutations to improve prediction accuracy, and the Bag of Bonds (BoB) descriptor, which encodes atomic interactions through bond-type-specific vectors and maintains permutation invariance.^64^ For periodic systems, extensions such as the Sine matrix and Ewald-summation matrix further improve scalability and accuracy by incorporating lattice periodicity.^65,66^

#### 2.3.2 Electronic structure representations

Electronic structure representations encode quantum mechanical properties of materials – energy levels, wavefunctions, and their momentum dependencies – into formats suitable for DL training and further computational analysis. This emerging field is particularly important for 2D materials, where reduced dimensionality often leads to distinctive electronic behavior and strong correlations that require specialized representation methods.

**Fig. 2:**
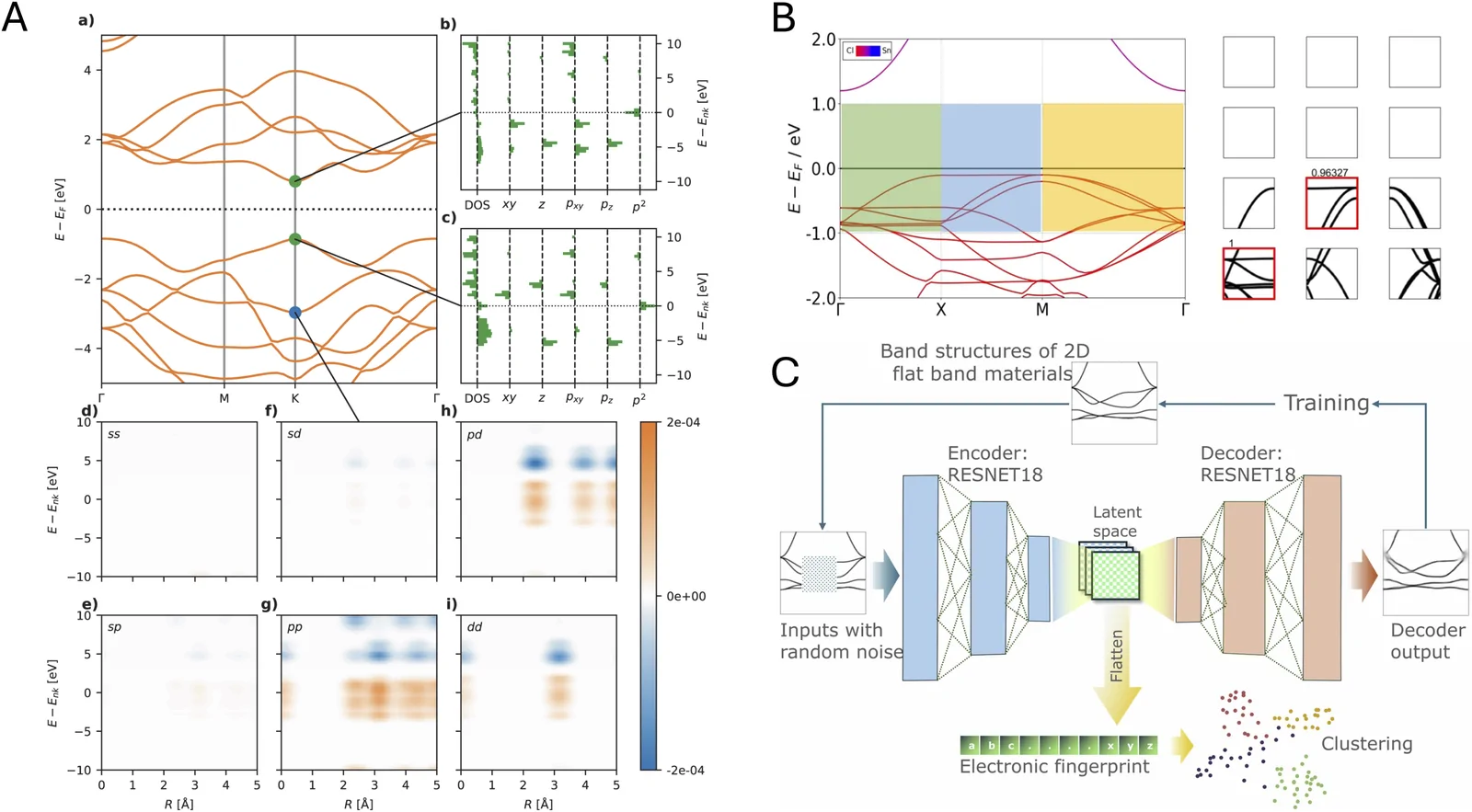
Electronic structure representations. (A) Energy Decomposed Operator Matrix Elements (ENDOME) and Radially Decomposed Projected Density of States (RAD-PDOS) fingerprints, adapted from ref. 67 with permission from Springer Nature,^67^ copyright 2025. (B) Segmentation of band structure images, adapted from ref. 68 with permission from Springer Nature,^68^ copyright 2025. (C) Convolutional autoencoder (CAE) elf, adapted from ref. 69 with permission from Springer Nature,^69^ copyright 2025.

The GW approximation calculates accurate quasiparticle energies by combining the single-particle Green's function (G) with the screened Coulomb interaction (W) to correct mean-field energies with many-body effects, but requires substantial computational resources. To bypass this, Knøsgaard *et al.* used DFT derived electronic structure descriptors as a starting point as DFT states encode materials structure well. They have designed two electronic descriptors *viz.* Energy Decomposed Operator Matrix Elements (ENDOME) and Radially Decomposed Projected Density of States (RAD-PDOS)^67^ to predict GW corrections from DFT calculations (see Fig. 2A). ENDOME first creates projections of position, momentum, Laplacian operators of a reference state with other states. Subsequently, it bins these projections based on energy differences of the states onto a Gaussian energy grid, producing a 6 × 50 fingerprint of energy-dependent features. Complementarily, RAD-PDOS constructs a correlation function in energy and radial distance, encoding DOS across different atomic orbitals into a 25 × 20 energy–distance grid that preserves orbital-specific electronic distributions. Thus, RAD-PDOS contains the information of environment of orbitals in Hilbert space. Finally, the concatenated fingerprints are passed into an XGBoost regression algorithm to train *G*_0_*W*_0_ energy corrections for each eigenvalues. Such physics-motivated approach enables accurate prediction of many-body effects at a fraction of the computational cost of traditional GW calculations, achieving mean absolute errors as low as 0.14 eV for electronic states in 2D materials.

Bhattacharya *et al.*^68^ pioneered segmentation techniques for band structure images, by dividing them into energy strips (of 0.5 eV each) and along high-symmetry k-paths and applying a supervised convolutional neural network (CNN), to identify flat bands, Fig. 2B. This approach overcomes the limitations of parameterized band structures, which often miss important electronic features due to band crossings and complex dispersions. Their CNN achieved high accuracy in detecting flat bands from segmented images without relying on arbitrary bandwidth definitions. Building on this, Pentz *et al.*^69^ developed elf (electronic fingerprint), a convolutional autoencoder framework with RESNET architecture that encodes band structure images into 98-dimensional fingerprint vectors, Fig. 2C. By training the model to reproduce electronic band structures within ± 4 eV around the Fermi level (even when portions were artificially obscured during training), elf effectively captures essential electronic patterns and creates meaningful fingerprints that cluster materials with similar band structures, revealing chemical and electronic relationships that traditional analysis methods had overlooked. Specifically, elf was able to group chemical compounds with similar stoichiometry by using their similarity in band structures, and was also able to identify duplicate entries in the 2D materials encyclopedia database autonomously.

#### 2.3.3 Hybrid representations

Hybrid representations integrate multiple data modalities – such as graph-based representations and text-based descriptors of physical properties – into a cohesive framework for deep learning. By combining local structural interactions (*e.g.*, atomic arrangements) with global electronic characteristics (*e.g.*, bandgap), these representations excel in tasks that require multi-objective optimization, like predicting stability and performance simultaneously.

The relationship between structural and electronic representations is vital to 2D materials research. The crystal structure, which defines how atoms are arranged, directly influences the distribution of electronic states that govern material functionality. Combining these representations in ML has been shown to improve prediction accuracy. Wang *et al.*^70^ developed a feature engineering strategy that constructs seven element-specific feature matrices from 2D material structure graphs. By processing these matrices *via* mean-pooling, they can create adaptive descriptors and select property-specific matrices through performance ranking to capture both structural topology and elemental information.

Recent advancements have further expanded the hybrid representations by incorporating diverse data sources. These include graph-based features derived from crystal structures, physical property measurements, and text-derived insights mined from scientific literature. Such comprehensive descriptors could enable richer understanding of 2D materials. For example, MatSciBERT^71^ utilizes transformer architectures to distill electronic and structural insights from vast materials science literature, offering a scalable approach to knowledge extraction. Additionally, MatText^72^ provides a benchmarking framework designed to evaluate and enhance text-based representations, focusing on predicting numerical properties from textual inputs.

### 2.4 Tools for data processing and mining

General high-throughput tools for navigating and analyzing materials databases are essential for accelerating ML applications in materials science, addressing challenges like duplicate structure identification, feature extraction, and materials space navigation. Efficient data processing and organization are prerequisite for applying DL for large datasets in 2D materials research. A range of tools and methods have been developed to extract features, ensure data consistency, and enhance ML model training by filtering redundant or inconsistent information, Table 2.

**Table 2:** Overview of tools for 2D materials research

Tools	Description	Link
Matminer	A python library for extracting and analyzing materials data, useful for feature engineering in machine learning studies of 2D materials	https://github.com/hackingmaterials/matminer
Pymatgen	A robust tool for materials analysis, enabling structure manipulation, property calculation, and simulation of 2D material systems	https://pymatgen.org/
Matbench	A benchmarking platform for machine learning models, providing datasets and tasks relevant to predicting 2D material properties	https://matbench.materialsproject.org/
Optimade	An API standard for querying materials databases, facilitating access to 2D materials data across repositories	https://www.optimade.org
MLMD	A machine learning framework for molecular dynamics, applicable to simulating and studying 2D material behaviors	https://github.com/Jiaxuan-Ma/MLMD
AlphaMat	A platform for computational materials design, offering tools to explore and optimize 2D material structures and properties	http://www.aimslab.cn
Constructor platform	A modular software suite for materials modeling, supporting simulations and workflows for 2D materials research	https://docs.constructor.tech/home/en-us/

Matminer automates the extraction of multiple descriptors – spanning electronic, structural, and thermodynamic properties – converting raw data into machine-readable formats.^73^ It supports high-throughput workflows by automatically fetching relevant descriptors and organizing them to input into models like CNNs and hybrid architectures. Pymatgen offers robust tools for retrieving and pre-processing data, including atomic positions, crystal structures, band gaps, *etc.*, by interfacing with large databases like Materials Project.^74^ It is widely used to construct graph-based representations of 2D materials, which serve as inputs for GNNs. Matbench provides standardized datasets for tasks like band gap or formation energy prediction.^75^ It also hosts a dataset of experimentally observed band gaps. Apart from providing standardized datasets, Matbench-discovery^76^ also provides a leaderboard which show the current best machine learning potentials. Jarvis-leaderboard is another source of standardized datasets including accurate data from quantum calculations, experimental superconductivity transition temperature, and interatomic potentials.^77^ These benchmarking datasets are essential for model validation, allowing consistent comparisons across different architectures and algorithms. These well-curated benchmarks ensure reproducible and generalizable model performance, helping researchers optimize hyperparameters and assess algorithm robustness.^78^

In addition to these tools, high-throughput workflows require addressing data duplication and inconsistencies, which often arise from variations in cell parameters or small perturbations in atomic positions within the accuracy of DFT. Isometry-based comparisons have been developed to detect duplicates robustly, ensuring database integrity and improving ML model reliability by avoiding redundancy.^79^ Metrics like the Local Novelty Distance (LND) further quantify deviations in structure similarity using continuous descriptors, enabling efficient navigation of materials spaces. Advanced tools such as Predicted Fraction of Improved Candidates (PFIC) and Cumulative Maximum Likelihood of Improvement (CMLI) have also been developed to directly assess the quality of design spaces, helping researchers prioritize regions with higher discovery potential.^80^

The development of integrated workflows for seamless data exchange and collaboration across domains is also worth noting. A few recent examples include: OPTIMADE^81^ application programming interface provides standardized access across multiple databases, acting as an enabler for AI-driven materials discovery; MLMD platform^82^ is dedicated to the integration of experiments, computation, and design of novel materials; AlphaMat platform^83^ aims at uniting materials science and AI; and the Constructor Platform is designed to simplify and accelerate the scientific research lifecycle.^84^ Such integrated workflows promise to speed up materials discovery to meet our increasing technological requirements.

## 3 Forward design with DL for properties prediction

Forward design refers to predicting material properties from its known atomic structure. DL has transformed this process by providing data-driven insights for electronic structures and their derived properties. Unlike traditional computational approaches that rely on first-principles calculations, DL offers accelerated prediction pathways while maintaining comparable accuracy, enabling more efficient exploration of material design spaces.

**Fig. 3:**
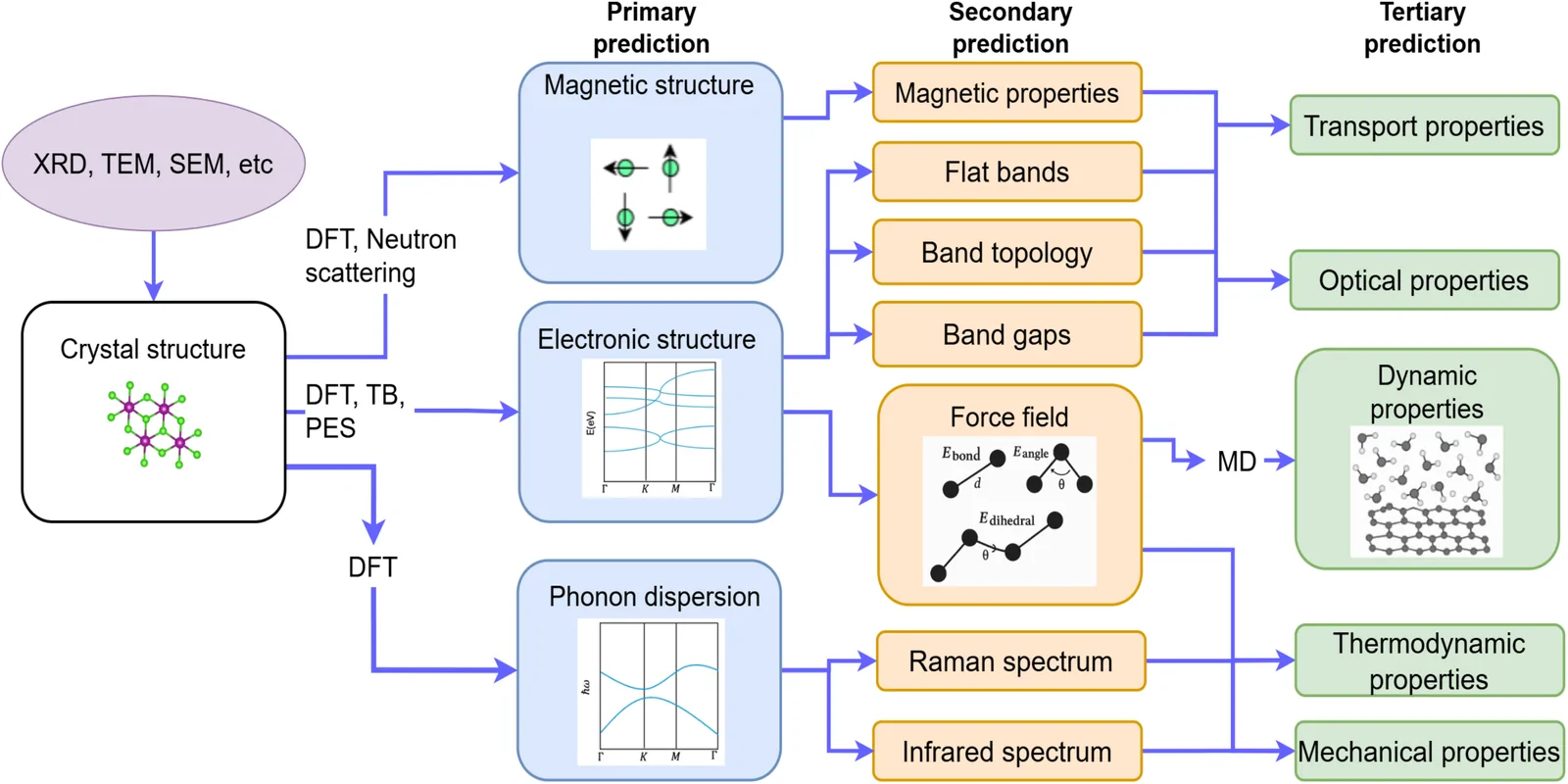
Forward design pathways for 2D material crystal structures to properties. Crystal structures are validated using experimental techniques like XRD, TEM *etc.* First principles simulations like DFT calculates electronic, phononic, and magnetic structure which in turn yields end properties like optical, transport, mechanical and dynamic properties.

This forward design workflow can be systematically divided into three primary levels of prediction,^85^ also see Fig. 3 for details. First, DL can model fundamental electronic quantum mechanical properties by emulating underlying numerical methods, such as DFT or tight binding (TB). Here, DL models mimic DFT-like methods and use generative algorithms to predict the electronic structure or phonon frequencies on a discretized grid. Second, DL can predict derived single-point outputs – energy gaps, total energy, or interatomic forces – effectively replacing computationally expensive DFT calculations. These predictive models serve as machine learning force fields (MLFF), enabling rapid molecular dynamics simulations. Finally, DL can analyze outputs from these simulations to predict measurable material properties such as thermoelectric coefficients, superconducting transition temperature, or photoelectric efficiency, to name but a few. In some advanced implementations, DL frameworks can even directly correlate atomic configurations to these physical properties, bypassing intermediate calculations entirely.

Beyond these general approaches to forward design, specialized DL architectures have been developed to incorporate physical knowledge and handle multiple objectives simultaneously. Physics-aware Neural Networks (PNNs)^86^ represent a specialized DL approach that explicitly embeds physical laws into neural network architectures. By incorporating known physical principles like symmetry considerations or conservation laws, PNNs excel in solving governing equations with sparse training data. Although their application to 2D materials' electronic structures remains limited, they show significant potential for modeling band structures or carrier dynamics in these systems.

Multi-Objective Optimization (MOO), enhanced by DL, enables the simultaneous optimization of multiple competing properties. MOO produces Pareto-optimal solutions representing the best possible trade-offs between different properties. When applied to 2D materials, this approach could optimize multiple properties simultaneously, such as electronic band gap and thermal conductivity, leading to more targeted material designs.

Although direct examples of PNNs and MOO applications in electronic structure prediction for 2D materials remain scarce^87^ – likely due to the complexity of quantum mechanical modeling and limited available datasets – the success of these methods in related fields suggests substantial untapped potential. This section explores the application of diverse DL approaches for forward design, examining methods for predicting fundamental electronic structures, applications for topological properties and strong correlations, prediction of flat bands and other quantum phenomena, and downstream property prediction critical to functional applications of 2D materials. Through these areas, we examine how DL is transforming our ability to predict and understand the complex electronic behaviors of 2D materials.

### 3.1 DL for predicting electronic structure

The prediction of electronic structures with DL can be approached in two fundamental ways. The first approach involves training neural networks to solve the underlying quantum many-body problems, specifically the electronic structure problem based on the many-electron Schrödinger equation or its approximations. In this physics-aware approach, the model outputs the electronic wavefunctions or electron density directly. The second approach focuses on directly predicting specific electronic properties – such as band dispersion, band gaps, or DOS – from crystal structures, effectively bypassing the computational complexity of quantum computations at the cost of reduced theoretical versatility. Detailed descriptions of these approaches are provided below and in Table 3.

#### 3.1.1 First-principles DL for electronic structure calculations

Calculation of electronic structure of real materials with DL is hard because many electrons interact with each other while obeying the Pauli principle, so their shared wavefunction must be antisymmetric and capture subtle correlations. Broadly, three principal strategies have emerged. The first strategy models the electron–electron interaction with a DL model. The second tackles the full many-electron problem by directly optimizing a flexible, physics-informed trial wavefunction with Variational Monte Carlo (VMC).^88^ The third strategy starts from density functional theory (DFT), and then improves DFT for strongly correlated regions *via* DL-assisted quantum embedding. In the following paragraphs, we discuss these methods in further detail.

**Fig. 4:**
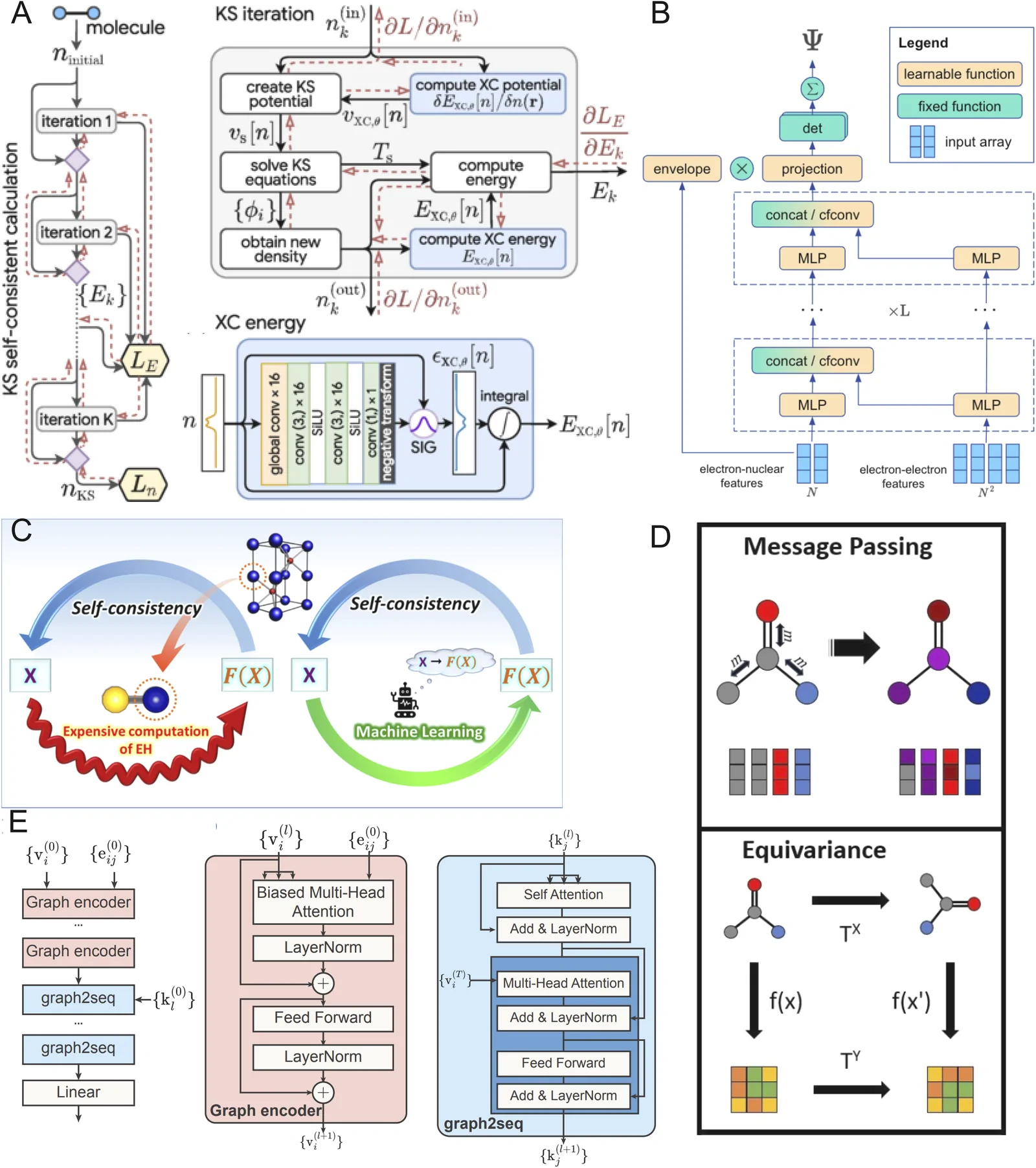
DL architectures (panel A, B and E) and techniques such as DL-assisted quantum embedding, message passing and equivariance for electronic structure prediction. (A) Solving Kohn–Sham equation with exchange-correlation energy estimated with DL, adapted from ref. 91 with permission from American Physical Society,^91^ copyright 2025. The left image show the iterative Kohn–Sham scheme, in which each iteration is divided into step shown in the right. The xc-energy term is modeled using the convolutional network described in the bottom right of panel A. (B) Architecture of Ferminet (adapted from ref. 95 with permission from *arXiv*,^95^ copyright 2025) shows L serial composite layers each made from a couple of MLP and convolution layers. (C) Comparison of the traditional quantum embedding path (left) with ML assisted calculation of embedding Hamiltonian in DL assisted quantum embedding calculations, adapted from ref. 108 with permission from American Physical Society,^108^ copyright 2025 (right) (D) Message passing (top) within a graph updates the nodes from neighboring node information. Equivariance (bottom) in GNN ensures the embedding transforms the same way as the input structure, adapted from ref. 113 with permission from Nordic Machine Intelligence,^113^ copyright 2025 (E) Graph based transformer network, Bandformer architecture graph encoder and graph2sequence modules. It is used for predicting band structure, adapted from ref. 114 with permission from *arXiv*,^114^ copyright 2025.

DFT handles many-body physics by approximating electron–electron interactions through exchange-correlation potentials. Several studies have demonstrated AI's effectiveness in modeling these exchange-correlation interactions,^12,89–91^ see Fig. 4A for details of one such architecture. This approach extends beyond standard DFT, enabling models to emulate advanced methods such as hybrid functionals and meta-generalized gradient approximation (*meta*-GGA) calculations, which provide a more accurate solution for exchange-correlation effects.^18,92^

Solving the complete many-electron Schrödinger equation presents a more formidable challenge, as it requires proper treatment of wavefunction anti-symmetry and complex electron–electron correlations. A particularly successful approach is using the Variational Monte Carlo method (VMC) technique which is a DL assisted Quantum Monte-Carlo method. In this probabilistic approach, a trial wavefunction respecting Pauli anti-symmetry and correlation is assumed and a variational method is used to reach the ground state. Hermann *et al.*^93^ introduced PauliNet, a DL framework using first-quantization representation that achieves nearly exact solutions for strongly correlated systems containing up to 30 electrons. In parallel, Pfau *et al.*^94^ developed FermiNet (see Fig. 4B for its architecture), another approach which uses first quantization to solve the many-electron Schrödinger equation. Both PauliNet and FermiNet use VMC with Slater–Jastrow–backflow (SJB) ansatz/representation for the wavefunction which consists of the Slater determinant for Pauli exclussion, Jastrow Factor for the short-range electron–electron correlation and backflow transformation to accurately incorporate correlation. More recently, von Glehn *et al.*^95^ introduced Psiformer, which replaces conventional neural networks in these models with transformer architecture, to better capture long-range electron interactions and improve convergence. While PauliNet, FermiNet (Fig. 4B), and Psiformer differ in their specific neural network architectures, they all employ VMC techniques to optimize electronic wavefunctions toward the ground state.^96^ Other ansatz/representations of quantum state wavefunctions which can be directly modeled as neural networks come under the umbrella of Neural Network Quantum States (NNQS) modelled with Restricted Boltzmann Machines (RBM)^97^ architecture. RBMs are probabilistic generative neural networks consisting of visible input and output layers and hidden layers for latent representation, and are trained to reconstruct the original distribution, thereby generative. An extension of RBMs called the deep Boltzmann machine^98,99^ was the first few architectures used as NNQS. Later, CNNs^100^ and autoregressive models^101^ were also used as neural networks modeling wavefunctions. Specifically, the attention mechanism within the autoregressive methods, *e.g.* transformer or Recursive neural networks, can automatically encode quantum correlations.^102,103^ All these networks are also optimized to reach the ground states using VMC.

Numerical techniques like DFT decompose the total energy of an electron to separate out the electron–electron interaction as exchange-correlation term, and solves the total energy by approximating this term. For strongly correlated systems, this assumption does not capture the interaction very well and we need special techniques^104^ in which we embed regions of strong correlations (where exact Hamiltonians need to be solved) within a region of weakly correlated systems where approximations works. Examples of such quantum embedding theories are Dynamic Mean Field Theory (DMFT), Density Matrix Embedding Theory (DMET), Quantum Defect Embedding Theory (QDET), Gutzwiller Approximation (GA) *etc.* DMFT uses a iterative frequency-dependent Green's function, which treats strong correlation as impurity within a dynamic bath of electrons, thus capturing dynamic effects *e.g.* phase changes. In contrast, DMET^88^ performs a computationally cheaper, frequency-independent self-consistency on the local density matrix, making it more efficient for ground-state properties *e.g.* energy. QDET^105^ is an embedding method using many-body perturbation theory to derive an effective Hamiltonian for localized defects, focusing on their ground and excited states. The Gutzwiller Approximation (GA),^106^ uses variational principles to provide a simplified wave function approach by suppressing double occupancy, offering a computationally inexpensive way to estimate correlation effects. Most of these approaches use expensive calculation of an embedding Hamiltonian making them almost an order of magnitude slower than traditional DFT. Almost a decade back, application of ML for replacing these computationally expensive steps were predicted to be feasible.^107^ However, application of DL in quantum embedding remains a handful. Rogers *et al.* have found an computational framework that replaces calculation of the expensive embedding Hamiltonian in a range of quantum embedding methodologies using a DL step, thereby reducing the computational cost to merely DFT level^108^ as shown in Fig. 4C. DL versions of these embedding techniques were also used for accurate interatomic force calculation in presence of string correlations. Suwa *et al.* have demonstrated a DL equivalent of a GA to carry out molecular dynamics showing 10^6^ times performance improvement over traditional^109^ quantum calculations. Structural dynamic studies in f and d-electron correlated systems have been carried out with a combined quantum embedding technique and DL based interatomic potentials. The interatomic potential is trained on DFT data with GA.^110^ Lately, NNQS has also been used in combination with DMET to model strongly correlated systems.^111^

Zheng *et al.*^112^ developed AIQM1, an AI-enhanced quantum mechanical method that combines semi-empirical calculations with DL and dispersion corrections. This hybrid approach achieves coupled-cluster level accuracy for a range of properties, including ground-state energies for complex compounds like fullerenes, while maintaining computational efficiency comparable to semi-empirical methods. Such capabilities make AIQM1 particularly valuable for studying 2D materials with delocalized electrons and complex electronic structures.

#### 3.1.2 Physics-aware DL models for electronic structure predictions

While first-principles DL models directly solve quantum mechanical equations, physics-aware models incorporate physics into their architecture without explicitly solving the Schrödinger equation. These models encode physical intuition through their design, ensuring better generalization and interpretability.

Building on the graph-based representations introduced in Section 2.3.1, message-passing layers of GNNs have proven particularly effective for electronic structure predictions.^53,115^ They propagate information through crystal graphs as shown in Fig. 4E, by iteratively updating atomic features based on their local chemical environments, effectively capturing quantum–mechanical interactions between atoms. Recent advancements include MGNN (Moment Graph Neural Network), which uses moment representations to capture spatial relationships between atoms while maintaining rotational invariance.^116^ Unlike many equivariant models (see below) that process tensor information throughout the entire network, MGNN contracts moment tensors to scalars at the beginning of message passing, making it computationally efficient while accurately predicting properties like energies, forces, dipole moments, and polarizabilities. This approach allows MGNN to handle complex systems with accuracy approaching traditional electronic structure methods but much greater computational efficiency.

An increasingly popular approach among researchers is the adoption of equivariant neural networks (ENNs). These networks are designed such that their outputs transform predictably under symmetry operations – such as translation, rotation, or inversion – enabling them to inherently respect the underlying symmetries of the physical system (see Fig. 4D for example). By embedding these invariance properties, ENNs reduce model complexity, decrease the demand for extensive training data, and enhance both prediction accuracy and physical consistency. Notable applications include improved electronic density predictions.^117,118^

A tensor-based DL model, OrbNet-Equi, developed by Qiao *et al.*,^119^ incorporates geometric data by enforcing equivariance under symmetry operations. The model shows promising results for predicting electronic structures of complex molecules. In a complementary study, Tang *et al.*^120^ introduced DeepH-hybrid, an E(3)-equivariant neural network designed to learn hybrid functional Hamiltonians as a function of material structure. By bypassing the computationally intensive self-consistent field iterations of traditional methods, DeepH-hybrid achieves accuracy comparable to conventional hybrid functionals, demonstrating its effectiveness in predicting electronic structures for large-scale 2D moiré supercells, such as twisted bilayer graphene. Knøsgaard *et al.*^67^ developed a gradient boosting (GB) model using physics-aware ENDOME and RAD-PDOS fingerprints (as detailed in Section 2.3.2) to predict non-self-consistent or one-shot *GW* (*G*_0_*W*_0_) band structures for approximately 700 nonmagnetic 2D semiconductors from the C2DB database.^26^

#### 3.1.3 Data-driven DL approaches for electronic structure predictions

In contrast to physics-aware models, which incorporate explicit physical constraints, data-driven DL leverages statistical patterns from training data, providing a flexible framework for predicting electronic structures. Initial attempts to predict the electronic charge densities and wavefunctions from crystal structures using AI relied on datasets generated by conventional DFT. These early models, using rather simple feedforward or deep neural networks, tackled a fundamentally generative task despite their relatively simple architectures.^85,121,122^ A key limitation of DFT, however, is its computational cost for large systems, which restricts its scalability. To circumvent this, Fiedler *et al.*^123,124^ proposed training an AI model to learn electronic structures for small structural units with a large system at finite temperatures, subsequently integrating these predictions using a generative framework. Similarly, 2D heterostructures – conceptualized as assemblies of monolayers with various stacking orders and twist angles – have been modeled using this hybrid approach. Tritsaris *et al.*^125^ employed tight-binding models within an agent-based simulation framework, using prototype 1D materials and realistic 2D materials, to predict band structures of twisted bilayer MoS_2_ and multi-layer graphene moiré superlattices.

The prediction of electronic band structures has evolved significantly with transformer-based architectures. For instance, the model Bandformer^114^ treats mapping of the structural graph of a crystal to its electronic band structure as a language translation task (as shown in Fig. 4F, it uses graph encoder and graph2seq modules to make sequences from structure graphs), encoding local atomic environments and high-symmetry k paths to predict the band centers (mean values) and dispersions (deviations from the mean) of the electronic bands near the Fermi level. Tested on the Materials Project database, Bandformer achieves mean absolute errors of 72 meV for band centers and 84 meV for band dispersions. As the first end-to-end approach for direct crystal structure to band structure prediction, these results are promising. While visual comparison between predicted and DFT-calculated band structures shows the model captures general electronic features, some fine details important for property prediction remain challenging to be reproduced accurately.

In the context of a more modest task of bandgap prediction, a material descriptor was developed for hybridized boron–nitrogen graphene with various supercell configurations, enabling DL models such as CNNs with transfer learning to capture the correlation between localized atomic clusters and the overall bandgap, achieving accurate bandgap prediction across different configuration scales.^126^

Zhang *et al.*^127^ evaluated four machine learning algorithms – support vector regression (SVR), multilayer perceptron (MLP), gradient boosting decision trees (GBDT), and random forest (RF) – for predicting bandgaps of 2D materials using C2DB. Their analysis revealed that GBDT and RF were the best for predicting bandgaps of 2D materials. Meanwhile, Rajan *et al.*^39^ focused on MXene materials, building a database of 7200 structures and applying LASSO (Least Absolute Shrinkage and Selection Operator) regularization to identify eight key features from an initial set of 47. Their Gaussian process regression model achieved exceptional accuracy with an RMSE (Root Mean Square Error) of 0.14 eV for bandgap prediction, enabling rapid screening of novel MXene compositions without requiring computationally intensive GW calculations.

Recently, transformer-based language models have been explored for predicting semiconductor band gaps by directly encoding material text descriptions. Yeh *et al.*^128^ demonstrated that the RoBERTa model can predict band gaps with high accuracy by processing textual representations of material properties, achieving a mean absolute error of approximately 0.33 eV. In a complementary approach, Lee *et al.*^129^ proposed CAST, a cross-attention based multimodal framework that fuses graph-encoded crystal structures with textual descriptions to predict material properties, showing improvements of up to 22.9% across multiple properties including band gap prediction.

In recent years, there have been concrete demonstrations of structure-to-band-structure prediction by machine learning models. Gong *et al.*^114^ introduced a graph-transformer framework that, given a crystal structure, predicts the full electronic band structure, including band gap, dispersion, and related features. Zhang *et al.*^130^ developed machine-learning models to predict the computed band gaps of double perovskite materials, illustrating progress in forward models for electronic properties. More recently, Wang *et al.*^131^ proposed a structure-informed framework for discovering flat-band two-dimensional materials, which combines a data-driven flatness score with multi-modal learning from atomic structures to identify topologically nontrivial flat-band candidates. These works underscore that while full accuracy remains a challenge, particularly for subtle features of band dispersion, AI models are increasingly capable of providing useful predictions beyond simple band gap estimates.

#### 3.1.4 Experiment-to-theory DL models for electronic structure predictions

DL has emerged as a powerful bridge between experimental measurements and electronic structure predictions, potentially revolutionizing how we extract quantum mechanical information from experiments. In crystallography, the fundamental ‘phase problem’ has long limited structure determination – conventional X-ray diffraction captures amplitude information but loses crucial phase data. Larsen *et al.*^132^ developed PhAI, a breakthrough approach combining CNNs with MLPs and a phase recycling mechanism to reconstruct complete electronic density maps at remarkably fine resolution, demonstrating how AI can overcome long-standing experimental limitations. For 2D materials, where sample quality and characterization challenges often arise, this approach offers promising pathways to structure determination from limited experimental data.

Beyond diffraction, other spectroscopic techniques have also benefited from DL-driven structure prediction. Vladyka *et al.*^133^ developed an MLP to analyze changes in X-ray emission spectra, focusing on Ge Kβ peaks at elevated pressures in amorphous GeO_2_. By encoding local atomic environments using Coulomb matrices, their model reliably predicts changes in coordination of a target atom from emission spectra, allowing for structural reconstruction from spectral moments.

Chen *et al.*^134^ trained their simple feedforward neural network with two hidden layers to predict ground-state electronic structures from core-loss spectroscopy. Using carbon K-edge ELNES/XANES spectra as input, their feedforward neural network with two hidden layers accurately reconstructed the carbon s- and p-orbital partial density of states (PDOS) for both occupied and unoccupied states. Their approach not only predicted electronic structures from experimental data but also demonstrated successful extrapolation to larger molecules, showing that noise-filtering preprocessing and careful model training enhance prediction performance for real experimental spectra.

**Table 3:** Summary of key DL methods for predicting electronic structure

Model type	Title of study	Structure representation	Summary	Target materials
First principles	PauliNet^93^	First-quantization, Slater–Jastrow-backflow wavefunction	Variational Monte Carlo (VMC) with DL ansatz, nearly exact for strongly correlated systems up to 30 electrons	Strongly correlated systems
	FermiNet^94^	First-quantization	Deep NN solves many-electron Schrödinger equation with antisymmetry, optimized by VMC	Strongly correlated systems
	Psiformer^95^	Transformer in first-quantization wavefunctions	Replaces conventional NN with transformer, better captures long-range electron interactions, improves VMC convergence	Correlated molecular systems
	Neural network quantum states (NNQS)^96,97,99^	RBM, DBM, CNN, autoregressive	Neural network wavefunctions trained with VMC; autoregressive transformers encode correlations efficiently	Generic quantum systems
	DL-assisted quantum embedding^108,109^	Embedding Hamiltonian	DL replaces costly embedding Hamiltonian step in DMFT/DMET/QDET/GA, reduces scaling to DFT level	Strongly correlated f/d electron systems
	AIQM1 (ref. 112)	Hybrid semi-empirical + DL + dispersion corrections	Achieves coupled-cluster accuracy with semi-empirical cost, effective for delocalized electrons	Complex compounds, fullerenes
Physics informed DL	MGNN (moment GNN)^116^	Crystal graphs, moment tensors	Efficient and accurate prediction of energies, forces, dipoles, polarizabilities	General crystals
	OrbNet-Equi^119^	Tensor + equivariant geometry	Enforces equivariance, improves accuracy in electronic property predictions	Complex molecules
	DeepH-hybrid^120^	E(3)-equivariant NN	Learns hybrid functional Hamiltonians without SCF with comparable accuracy	Moire supercells
	ENDOME/RAD-PDOS^67^	Physics-aware electronic fingerprints ENDOME and RAD-PDOS	Predicts G0W0 band structures for ∼700 semiconductors	Nonmagnetic 2D semiconductors
Data-based DL arroaches	Bandformer transformer^114^	Graph-to-sequence (structural graph > band sequence)	Treats band prediction as language translation; MAE ∼72 meV for band centers	Materials project crystals
	Basic CNN^126^	Local cluster descriptors	Captures bandgap variations in B–N graphene supercells	Hybridized B–N, graphene
	Gaussian process regression + LASSO^39^	Descriptors from materials properties	Predicts MXene bandgaps (RMSE 0.14 eV) after feature selection	MXenes
	RoBERTa (transformer, NLP)^128^	Textual materials descriptions	Language model predicts bandgaps from text (MAE ∼0.33 eV)	Semiconductors
	CAST (cross-attention multimodal)^129^	Graph + text fusion	Improves predictions (band gap + others) by combining structure + literature embeddings	General crystalline materials
DL for experiments	PhAI (CNN + MLP)^132^	Diffraction patterns + phase recycling	Reconstructs electron density maps, solves crystallography phase problem	2D crystals
	MLP (Coulomb matrices)^133^	Encoded local environments	Reconstructs structure from X-ray emission spectra under pressure	Amorphous GeO_2_
	Feedforward NN (2 hidden layers)^134^	ELNES/XANES spectra	Reconstructs carbon PDOS from spectroscopy, generalizes to larger molecules	Carbon-based materials

### 3.2 DL for interatomic potentials

Interatomic potentials capture empirical forms of interactions among species under various geometries. Traditional approaches fit forces and energies from first-principles simulations to predetermined functional forms, including simple empirical potentials (Lennard–Jones, Morse), bond-order potentials modeling directionality and variable bond strengths (Tersoff, REBO), and embedded-atom method potentials for metallic systems. These conventional fittings typically rely on least-squares methods or evolutionary algorithms such as genetic algorithms (GAs), which often struggle with complex structural configurations.

**Fig. 5:**
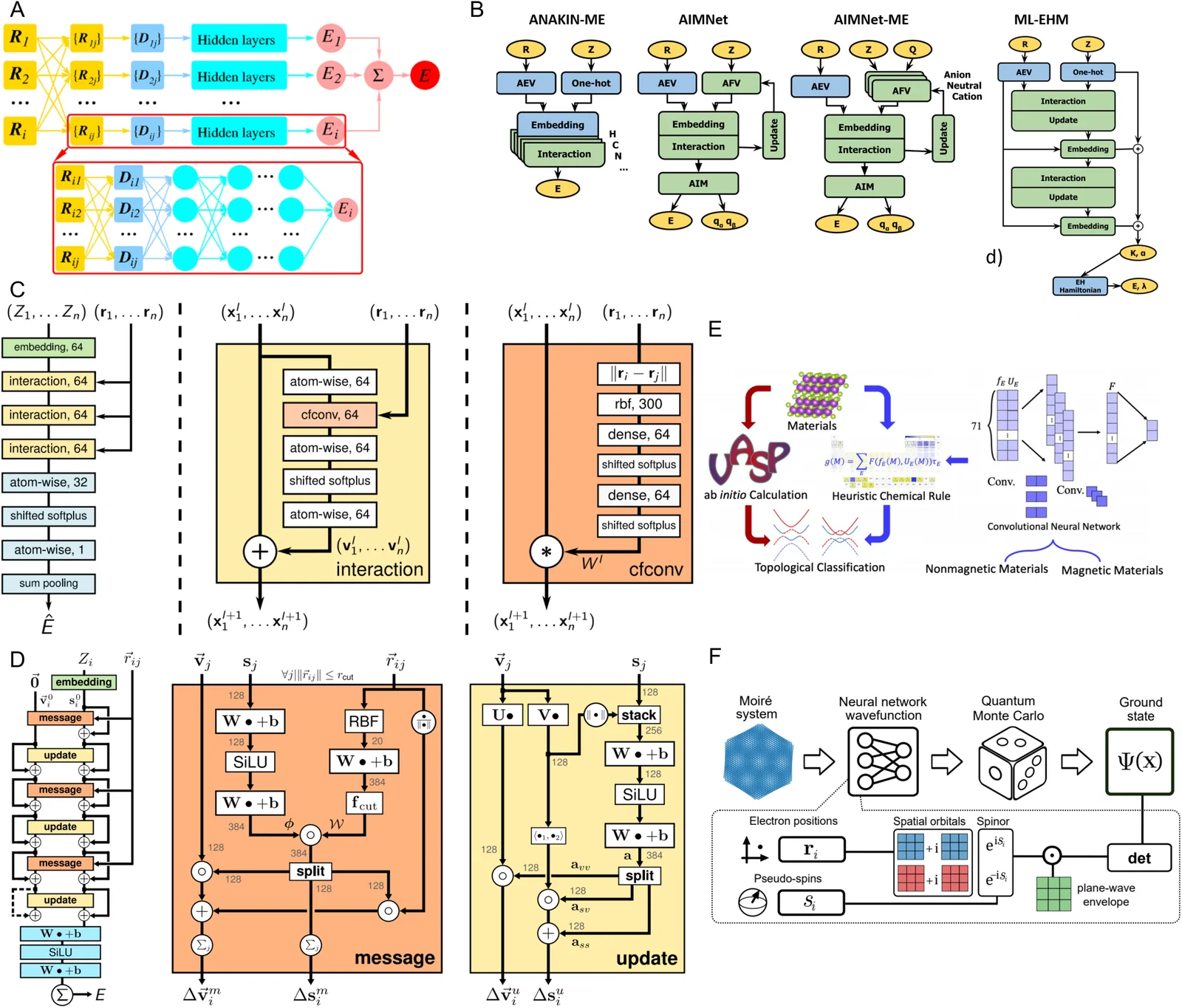
Various architectures used for generating interatomic potentials and identifying topological properties. (A) DPMD architecture, one of the first deep learning based interatomic potential, adapted from ref. 135 with permission from American Physical Society,^135^ copyright 2025. (B) Comparison of architectures of various high-dimensional NN potentials, adapted from ref. 136 with permission from American Chemical Society,^136^ copyright 2025. (C) SchNet architecture, a rotationally invariant interatomic potential, adapted from ref. 137 with permission from *arXiv*,^137^ copyright 2025. (D) PAINN architecture, one of the equivariant graph NN, adapted from ref. 138 with permission from *arXiv*,^138^ copyright 2025. (E) Topogivity pipeline, adapted from ref. 139 with permission from American Physical Society,^139^ copyright 2025. (F) Deep learning based identification of topological insulators, adapted from ref. 140 with permission from *arXiv*,^140^ copyright 2025.

DL has revolutionized this field by eliminating the need for predefined functional forms, instead learning the potential energy surfaces directly from data (Table 4). DL-based potentials can accurately model geometrical configurations, energy–distance relationships, and many-body interactions while reducing human bias in the fitting process.^141^ This approach allows for substantially improved accuracy and transferability across diverse atomic environments, particularly important for 2D materials with their unique bonding characteristics and surface effects. Various implementations have emerged, including DEEPMD^135^ (see Fig. 5A for its architecture), which uses deep neural networks to represent the many-body potential energy function, and ænet-PyTorch,^142^ which implements atom-centered neural networks with specialized symmetry functions. These ML potentials can achieve near-DFT accuracy at a fraction of the computational cost, enabling large-scale molecular dynamics simulations of 2D materials that would be prohibitively expensive with conventional *ab initio* methods.

Unlike conventional DL approaches that rely purely on data-driven optimization or generative frameworks, physics-aware neural networks integrate explicit physical laws – such as governing differential equations, conservation laws, or symmetry constraints – directly into their architectures or training procedures. Physics-informed neural networks (PINNs) represent a specific subset of these approaches, typically enforcing physical constraints through additional terms in the loss function. More broadly, physics-aware DL models include various architectures and methods designed to respect and incorporate physical insights beyond just loss-function regularization. This integration of data-driven learning and physical knowledge provides enhanced interpretability, improved accuracy, and physical consistency. Consequently, physics-aware models, including PINNs, are especially beneficial in scenarios involving limited data, constrained computational resources, or when predictions must rigorously adhere to fundamental principles, as commonly encountered in modeling complex systems like 2D materials.

One of the earliest attempts to make physically aware potentials was by encoding the local environment in the model, allowing it to choose a reference geometry close to the training examples during prediction. For example, PINN potentials were proposed to enhance the transferability of machine-learning interatomic potentials by combining a physics-based analytical bond-order model with neural network regression, showcased through a general-purpose PINN potential for aluminum and tantalum.^143–145^ Recently, it was found that in case of 2D materials, separating interlayer and intralayer interactions while modeling interatomic potential can lead to almost an order of magnitude rise in accuracy over potentials that treat all interactions together. The authors also found that for Moire lattices, a physics-aware validating metric based on stacking configurations performs much better than traditional metrics like force and energy.^146^

A challenge for any interatomic potential is to remain transferable for any geometric configuration, especially for NN-based potentials as they do not work from first principles. Towards this, Behler and Parrinello^147^ designed high-dimensional NN potential which calculates potential energy surfaces as superposition of atomic contributions. This allowed complex geometries to be modeled precisely using NN potentials. This also encodes the atomic structures as Atomic Environment Vectors (AEVs) and embeds the associated symmetries with Behler–Parrinello symmetry functions or Justin Smith symmetry functions.^136^ Several extensions of these high-dimensional potentials are lately designed *e.g.* ANAKIN-ME (ANI),^148^ AIMNet,^149^ AIMNet-ME,^150^ ML-EHM.^151^Fig. 5B gives a comparative picture of their architectures. Recently, further iterations of these potentials were reported with improved long-range interaction models with dispersion correction, electrostatic interactions, *etc*^152^. Another challenge for interatomic potentials is to remain relevant for various chemical environments. In last five years, several such Universal ML Inter-atomic Potentials (UMLIPs) were developed opening the door for creating new materials which are chemically stable.^153^ Even though UMLIPs are typically trained on extensive datasets covering diverse chemical and coordination environments, they often struggle with out-of-distribution predictions^154^ – for instance, accurately predicting surface energies, since surfaces inherently break periodic boundary conditions by definition.^155^ These potentials have not only shown promises in predicting evolving dynamic simulations, they show accurate prediction for even collective phenomena like phonon behavior.^156^ The first UMLIP was MEGNet^51^ which used a graph architecture and was trained on the Materials Project database. Since then several UMLIPs were developed which mostly used graphs to encode structure information.^15,157–160^ Clearly, graph NNs play a pivotal role in encoding wide range of chemical and coordinate information helping build UMLIPs. Therefore, we are going to discuss the various graph-based ML potentials in the coming paragraphs.

Graph NNs have been the representation of choice for physics aware potential development. Some of the graphs include physical awareness by encoding invariance to rotations. Examples of such GNNs are ALIGNN-FF,^161^ SchNet^137^(Fig. 5C), MEGNet,^51^ M3GNet^158^ and so on.

Similar to electronic structure prediction, equivariance principle has also been introduced for designing interatomic potentials as well to explicitly respect symmetries such as translational, rotational, and permutation invariance. Several popular interatomic potentials were generated around this equivariance principle. NequIP is an interatomic potential which employs message-passing GNNs and E(3)-equivariant convolution operations on tensors, thus requiring much less data for training and achieving accuracy of *ab initio* simulations.^162^ Several interatomic potentials were then developed based on message passing GNNs, *e.g.* EGNN,^163^ E2GNN,^164^ PAINN^138^ (Fig. 5D), GemNet,^165^ SEGNN,^166^ NewtonNet^167^ and many more. Allegro is another interatomic potential which does not use atom-centered message passing but makes a many-body potential using a tensor product of equivariant representations.^168^ Another message passing equivariant NN potential is MACE, which uses higher order messages to reduce the number of message passing iterations.^159^

The development of accurate UMLIPs has greatly accelerated simulations and property predictions of 2D materials, though significant challenges remain in ensuring their reliability and transferability across diverse conditions.^15^

### 3.3 DL for topology and strong electronic correlations

Topological materials represent a frontier in condensed matter physics, characterized by electronic properties that remain robust against perturbations due to the underlying mathematical principles. Over the past decade, systematic approaches have been developed to discover and classify topological band structures in condensed matter systems by considering both local symmetries and crystalline symmetries.^169–171^ A key feature of these systems is the topological obstruction that prevents smooth transitions between electronic states with different topological characteristics, requiring the closing of energy gaps or other dramatic changes in the electronic structure.

**Table 4:** Summary of DL methods for interatomic potentials

Model architecture	Title of study	Structure representation	Summary	Target materials
Deep NN based models	DEEPMD^135^	Atomic coordinates + environment descriptors	Deep NN represents many-body potential energy surface; achieves near-DFT accuracy for large-scale MD	2D + 3D materials
	ænet/ænet-PyTorch^142^	Atom-centered symmetry functions and descriptors	Training of neural network interatomic potentials on both energies and forces, using force with energy improves performance	Metallic and ionic systems
	PINN potentials^143,144^	Analytical bond-order + NN regression	Hybrid representation gives better transferability to unseen atomic configurations (defects, surfaces, compression, *etc.*); demonstrated for Al and Ta	General crystalline solids
	HDNNPs^147^ (*e.g.* ANI, AIMNet, AIMNet-ME, ML-EHM)	Atomic environment vectors (AEVs) + Behler-parrinello/Justin-Smith symmetry functions	High-dimensional NN potentials calculates energy as superposition of atomic contributions; handles complex geometries	Diverse chemical systems
Graph NN based models	ALIGNN-FF^50^	Graph + line graph (bond angles)	Physics-aware graph NN interatomic potential; explicitly includes bond angles	2D/3D crystals
	SchNet^137^	Continuous-filter convolution on atomic environments	Rotationally invariant NN potential, widely used as benchmark	Molecular + solid-state systems
	M3GNet^158^	Graph NN with 3-body interactions	Incorporates higher-order interactions, universal interatomic potential	Wide range including 2D materials
Equivariant representations	NequIP^162^	Message-passing GNN + E(3)-equivariant convolutions	Internal features that transform like tensors under rotation/translation. This requires far less training data to achieve *ab initio* accuracy	2D and 3D systems
	PaiNN^138^	Equivariant message passing with scalar + vector features	PaiNN can predict tensorial molecular properties (like dipole moments, polarizability) and simulate molecular spectra (IR, Raman)	Molecular & crystalline
	GemNet^165^	Equivariant graph-based encoding	Two-hop message passing to capture distances, angles, and dihedrals, invariant to translations, equivariant to permutations and rotations	Molecular & crystalline
	SEGNN^166^	Equivariant graph-based encoding	Node and edge features include physical quantities like vectors or tensors (*e.g.* forces, velocities). Uses steerable MLPs and equivariant message passing	Molecular & crystalline
	MACE^159^	High order E(3) equivariant message passing	Uses higher-order messages (up to four-body interactions) rather than only pairwise ones, reaches SOTA accuracy in low data regime	Molecular & crystalline
	Allegro^168^	Tensor product equivariant reps	Builds many-body potential without atom-centered message passing	Molecular & crystalline

The mathematical relationship between TB Hamiltonians and topological invariants has been rigorously established in condensed matter theory, providing a good foundation for ML applications. This precise correspondence has been explored by building supervised ML algorithms that learn mapping from TB parameters to topological properties without requiring explicit calculation of topological invariants.^172–174^

Complementing these supervised approaches, Scheurer and Slager^175^ demonstrated that unsupervised clustering techniques can also classify topologically distinct TB Hamiltonians. This approach is particularly powerful because it does not rely on specific parameterizations of the Hamiltonian. Taking a different direction, Peano *et al.*^176^ employed CNN to generate TB Hamiltonians directly from unit cell geometries, effectively capturing topological electronic features. This method leverages the NN to map arbitrary atomic structures to symmetry-enhanced TB models, enabling prediction of band structures and their topological properties, such as fragile topologies and Chern numbers, with high accuracy and computational efficiency. Extending this paradigm of leveraging ML for topological design, explicit topology optimization, utilizing the Moving Morphable Components (MMC) method as described by Du *et al.*,^177^ defines a structure descriptor, where a multitask learning (MTL) model concurrently predicts discrete-valued topological invariants and bandgaps for higher-order topological insulators.

As multiple DFT databases of 2D materials have been developed, ML tools mapping between realistic 2D material structures and topological structures were made possible. Schleder *et al.*^178^ employed the multi-task Sure Independence Screening and Sparsifying Operator (SISSO) method to engineer atomic feature-based descriptors from DFT databases, followed by the XGBoost tree algorithm to classify materials as topologically trivial or non-trivial with over 90% accuracy. This approach enabled the prediction of 56 novel topological materials, including 17 quantum spin Hall insulators, without requiring *a priori* structural knowledge, demonstrating significant advancement over traditional trial-and-error methods.

Building on this trend of ML applications in topological material discovery, Xu *et al.*^139^ introduced “topogivity”, a machine-learned chemical parameter that quantifies each element's tendency to form topological materials, enabling researchers to predict whether a material is topological based solely on its chemical formula with high accuracy (>80%). This DL architecture shown in Fig. 5E led to the discovery of new topological materials that could not be identified using traditional symmetry-based methods, demonstrating a simple and effective heuristic for materials discovery.

DL has proven to be valuable for phase classification in topological strongly correlated systems. For Fractional Quantum Hall (FQH) systems specifically, both supervised approaches, as demonstrated by Matty *et al.*^179^ or Li *et al.*^140^ (see Fig. 5F), and unsupervised methods, such as those employed by Jiang *et al.*,^180^ Jin and Wang,^181^ have been successfully applied to identify and characterize the complex phases in these systems. Zhang and Kim^182^ developed quantum loop topography, which constructs specialized input features for neural networks that successfully distinguish Chern insulators and fractional Chern insulators from trivial insulators. More recently, Teng *et al.*^183^ applied attention-based neural network-variational Monte Carlo methods to accurately predict wavefunctions in FQH systems, revealing microscopic features beyond traditional approximations. In a different approach, Noronha *et al.*^184^ demonstrated that neural networks can predict the Bott index (a topological invariant) in 2D topological superconductors with magnetic impurities by analyzing local DOS, providing an efficient method to identify topological phases from experimentally accessible measurements.

The presence of flat bands is an indicator of strong electronic correlations since suppression of kinetic energy enhances electron–electron interactions, leading to correlated quantum phenomena such as chiral plasmons,^185^ Chern insulators,^186^ and unconventional superconductivity,^2^ observed in twisted bilayer graphene and other 2D systems. Hence, high-throughput computational methods have been employed to identify flat bands in 2D materials.

Top-down data-driven searches leverage constraints such as bandwidth^187–189^ to screen materials using DFT calculations. These attempts suffer from arbitrariness in labeling band index due to band crossings. A CNN model was introduced to detect flat bands directly from images of electronic band structures, eliminating the dependency on band indexing.^68^ Through periodic table representations,^190,191^ recent studies employed CNN to predict occurrence of flat bands in 25 new Heusler alloys. Ma *et al.*^192^ have used a MLP to predict band gap in flat band system of twisted bilayer graphene (TBLG) with help of a physically interpretable descriptor designed with SISSO method. In a similar study on twisted bilayers dubbed DeepH, a DL model is used in predicting band gap and bandwidths of flat bands.^193^ Another study^194^ uses the DeepH model to explore MoSe_2_/WSe_2_ moiré lattices. Classification of flat band systems was achieved through an autoencoder based self-supervised model and subsequent clustering algorithms.^69^ In another research, a CNN is used to identify unique signatures of flat band states to distinguish them from conventional localized and extended states by training on wavefunctions from a molecular orbital representation.^195^

### 3.4 DL for other downstream tertiary properties

Beyond fundamental electronic structures, DL has successfully predicted numerous application-specific materials properties that directly inform practical applications. These downstream tertiary properties are crucial for identifying 2D materials that are suitable for specific technological needs.

In thermoelectric applications, several approaches have shown promising results. Gan *et al.*^196^ combined high-throughput DFT calculations with neural networks to accurately predict maximum *ZT* (dimensionless thermoelectric figure of merit that quantifies the efficiency of a material in converting heat to electricity), and optimal doping types in layered semiconductors. Na *et al.*^197^ introduced DopNet, which explicitly models host materials and dopants separately, achieving 68% lower prediction errors for unseen materials. Ishiyama *et al.*^198^ demonstrated the usage of Bayesian optimization to enhance thermoelectric properties of III-V semiconductor thin films, achieving a three-fold *ZT* improvement in just six optimization cycles. Beyond thermoelectrics, Wang *et al.*^199^ developed a self-supervised probabilistic model for shape memory alloys that learns atomic representations directly from crystal structure data, enabling the discovery of novel shape memory alloy candidates. Magnetic properties have also been successfully predicted using GNNs. Minch *et al.*^200^ developed a graph-based DL algorithm using ALIGNN^50^ model to predict atomic magnetic moments of 2D materials based on a Cr_2_Ge_2_Te_6_ prototype.

The Hierarchical Correlation Learning for Multi-property Prediction (H-CLMP) framework, as presented by Kong *et al.*,^201^ addresses the challenge of predicting multiple material properties simultaneously. Their approach integrates three key components: (i) composition-based property prediction, (ii) the learning of correlations among target properties within a multi-target regression framework, and (iii) the use of transfer learning to leverage training data from related but distinct property domains. The model was demonstrated by predicting spectral optical absorption coefficients across a range of photon energies for complex metal oxides, using only their elemental compositions. The best performance was achieved with the transfer learning extension, where a GAN was pre-trained on computational DOS data – a tangential property domain – and then employed to augment the prediction of absorption coefficients. This work shows how extra data can improve predictions when direct training data is limited.

## 4 DL models for inverse design of 2D materials

While forward design maps material structures to their electronic properties as reviewed in the previous section, inverse design addresses the more challenging problem: identifying material structures that yield desired electronic properties. This reverse mapping presents significant challenges because the forward relation is not one-to-one (injective) – multiple material structures (polymorphs) or conditions (temperature, pressure) can produce similar properties, and small structural variations can lead to dramatically different electronic behavior.

Data-driven inverse mapping from the property/functional space to the chemical space^202^ has evolved substantially in recent years, transforming from traditional search-based approaches to sophisticated generative AI methods. This evolution represents a paradigm shift in how we conceptualize materials discovery, moving from discrete sampling to continuous exploration of chemical space.

**Fig. 6:**
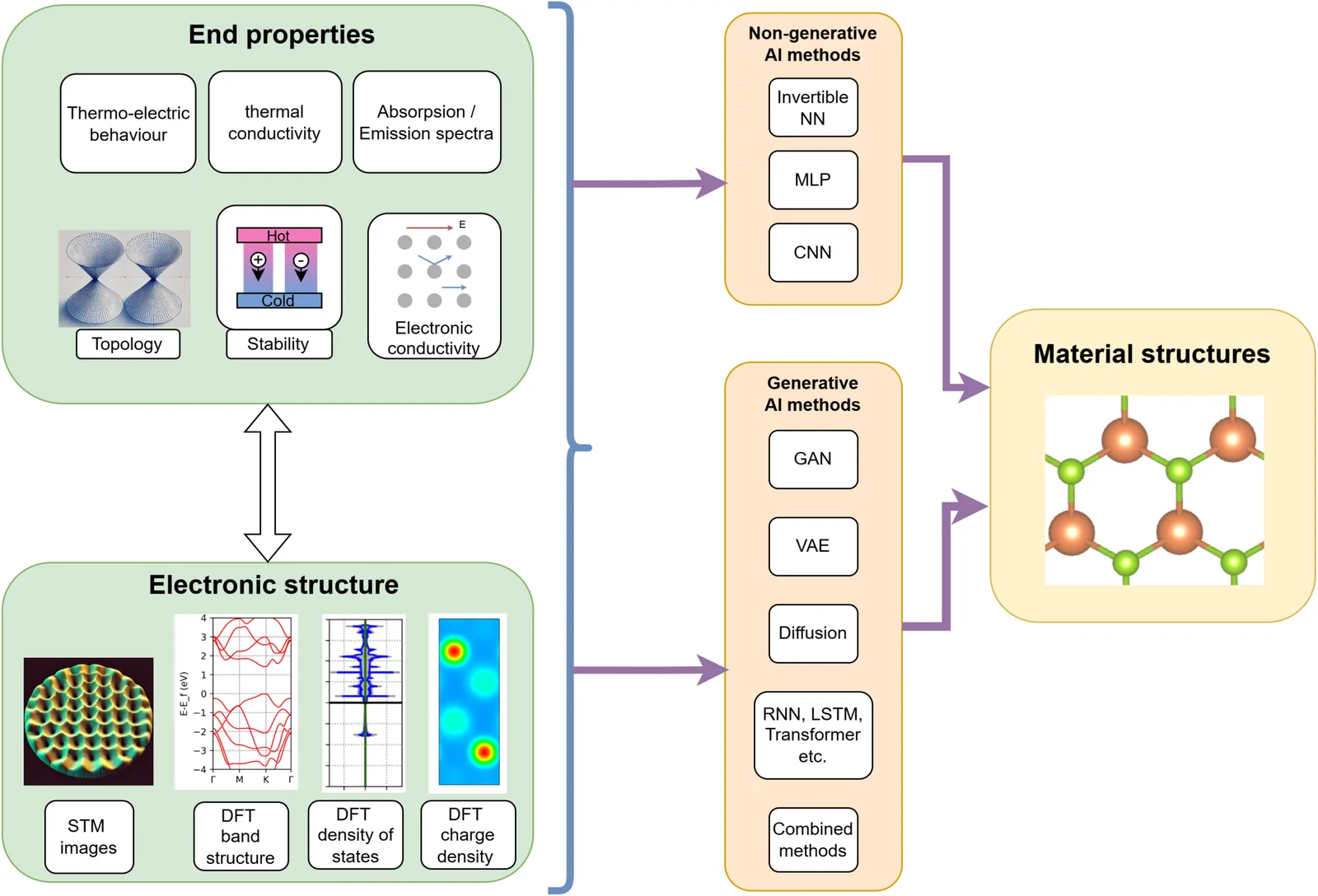
Inverse design using DL to identify material structures can start from either desired electronic structures or end properties of the materials. The DL architectures can be broadly classified into non-generative and generative categories. Examples of non-generative model architectures are dense or convolutional NNs or invertible NNs, whereas examples of generative architectures are GANs, VAEs, diffusion and autoregressive models.

Inverse design approaches can be broadly categorized into two main frameworks: non-generative and generative methods. Non-generative approaches include: (a) high-throughput screening of discrete chemical space to locate the desired material candidate, (b) evolutionary algorithms such as genetic algorithms, particle swarm optimization, Monte Carlo tree-search, and random walk based materials design, and (c) iterative optimization techniques including Bayesian optimization (BO) and reinforcement learning (RL).^13,203^ The DL architectures behind these approaches are illustrated in Fig. 6. In these non-generative approaches, models identify optimal candidates with desired properties from an existing pool of materials.

In contrast, generative architectures – including VAE, GAN, autoregressive models, and diffusion models, as shown in Fig. 6 – represent a fundamental shift, as they learn to generate entirely new material candidates within a continuous chemical space.^202–205^ Rather than searching existing databases, these methods learn the underlying distribution of valid materials and can generate novel structures that may not exist in the training datasets but possess target properties.

For 2D materials specifically, inverse design presents unique opportunities and challenges. The reduced dimensionality and distinctive quantum confinement effects of 2D systems create electronic properties highly sensitive to structural modifications, making them particularly suitable targets for AI-driven design. In this section, we examine both non-generative and generative DL approaches for inverse design of 2D materials, their implementation strategies, and the challenges they face.

### 4.1 Non-generative DL for inverse design of 2D materials

While traditional inverse material design approaches like evolutionary algorithms often operate without neural networks, modern implementations increasingly incorporate DL to enhance their efficiency and performance. This section focuses specifically on neural network-assisted inverse design methodologies, including decision tree frameworks, direct mapping networks, invertible neural networks, and optimization-enhanced approaches.

#### 4.1.1 Neural network-enhanced high-throughput search

Neural networks can serve as powerful classifiers within decision tree frameworks,^210^ enabling more complex decision boundaries than conventional trees. One of the classification-based material design platforms is Machine Learning for Material Design (MLMD).^82^ It includes multiple non-generative AI algorithms for classification and regression, *e.g.* modules for Support Vector Machines (SVM), RF, logistic regression, K-nearest neighbor regression, Catboost regression, *etc.* It also hosts surrogate optimization modules for GAs, differential evolution, particle swarm optimization, simulated annealing, and NSGA-II and active learning modules for Bayesian optimization.

**Fig. 7:**
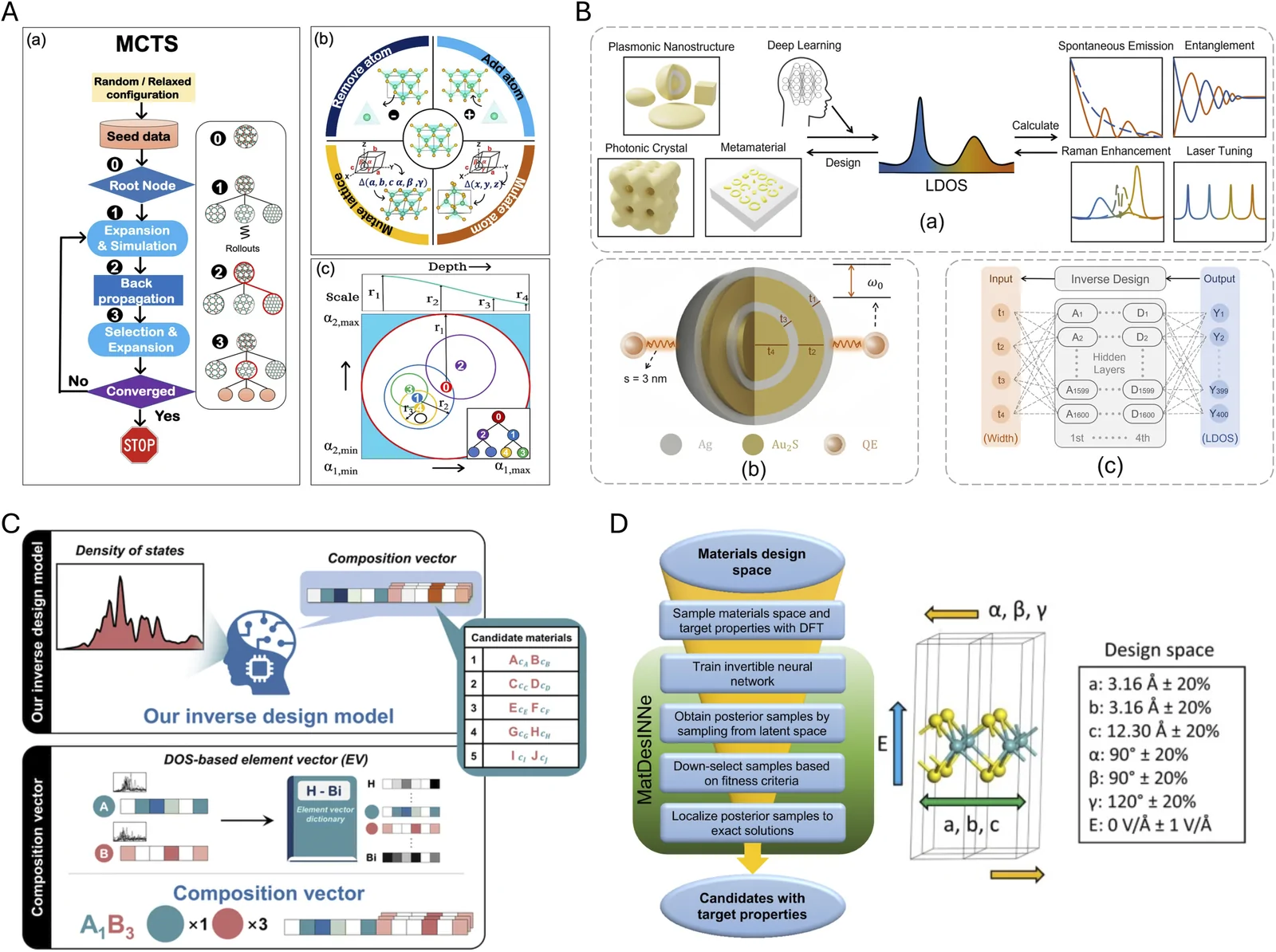
Non-generative inverse deep learning. (A) CASTING: a Continuous Action Space Tree search framework for inverse design, adapted from ref. 206 with permission from Springer Nature, *npj Comput. Mater.*, 2023. (B) Inverse design in quantum nanophotonics *via* LDOS-guided deep learning, reproduced from ref. 207 with permission from Walter de Gruyter/Science Wise Publishing, *Nanophotonics*, 2023. (C) Deep-learning inverse design model predicting compositions from the target DOS, adapted from ref. 208 with permission from American Chemical Society, *J. Mater. Chem. A*, 2024. (D) Inverse materials design workflow and parameter-space specification for MoS_2_, reproduced from ref. 209 with permission from Springer Nature, *npj Comput. Mater.*, 2021.

A notable advancement in decision tree frameworks is CASTING^206^ (see Fig. 7A), which significantly enhances efficiency of decision making by introducing Monte Carlo Tree Search (MCTS) based on reinforcement learning. The framework has been used for predicting structures of representative examples of 2D materials – graphane and hexagonal boron nitride.

Combination of high-throughput experiments with active learning algorithm called Gaussian process BO have recently been used for designing quasi-2D halide perovskites by optimizing photoluminescence intensity and chemical stability.^211^

#### 4.1.2 Direct mapping neural networks

Feedforward and CNNs offer a straightforward approach to inverse design through direct mapping between property and structure spaces. Liu *et al.* demonstrated this approach as shown in Fig. 7B, using a fully-connected NN to predict local DOS as a forward design step and then applying gradient-based optimization to determine material structure during the inverse step.^207^

For more complex property–structure relationships, CNNs have proven particularly effective. Bang *et al.*^208^ used a CNN architecture to directly predict composition vectors (see. Fig. 7C) describing crystal structures from vector representations of DOS data, enabling the discovery of inorganic crystals optimized for catalysis and hydrogen storage applications. The CNN's ability to capture spatial hierarchies in data makes it well-suited for translating between electronic and structural representations.

In the domain of semiconductor heterostructures, Pimachev and Neogi^212^ developed a hybrid approach combining random forests and neural networks for forward prediction of electronic properties from crystal graph representations. For inverse design, they employed a CNN to map desired band structures back to corresponding heterostructure configurations, effectively establishing a bidirectional relationship between structure and properties.

#### 4.1.3 Invertible neural networks

Invertible neural networks (INNs) represent a specialized architecture particularly well-suited for inverse design problems. Unlike conventional neural networks, each layer of INNs are bijective, *i.e.* both injective (each distinct input has a distinct output) and surjective (every output must have at least one input). This bijective property is achieved through careful architectural choices that avoid information loss, such as eliminating pooling layers and ReLU activations, while implementing coupling layers – which divide the input into two parts, apply transformation to one part, and recombine to create invertibility – hence preserving information through reversible transformations.^213^

The bijective nature of INNs allows them to be trained in the forward direction (structure to properties) and directly applied to reverse (properties to structure) without additional optimization steps. MatDesINNe,^209^ shown in Fig. 7D, demonstrated this capability for 2D materials by developing an INN that predicts electronic bandgaps of MoS_2_ under varying conditions of tensile strain and applied electric field. Once trained, their model could directly generate combinations of strain and field values that would yield a target bandgap, providing a computationally efficient pathway for property-based materials design.

### 4.2 Generative DL methods for inverse design

Unlike non-generative approaches that search existing material spaces, generative DL methods create entirely new material candidates by learning the underlying distribution of valid materials. These approaches offer unprecedented opportunities for exploring the vast chemical space of 2D materials by generating novel structures with targeted electronic properties.

Generative AI models – including VAEs, GANs, diffusion models, and sequence-based models – have demonstrated significant impact in inverse materials design. As summarized in Table 5, these frameworks employ diverse architecture designs and structure embedding algorithms. We organize these approaches into five categories: (a) GAN-based approaches, (b) VAE-based approaches, (c) diffusion models, (d) RNN and transformer-based sequence models, and (e) hybrid approaches combining multiple generative AI models.

**Table 5:** Summary of recent works in inverse material design using generative AI

Model architecture	Title of study	Structure representation	Summary	Target materials
Cross domain GAN	CrystalGAN^214^ (Fig. 8A)	Simple matrices formed of lattice vectors and fractional coordinates	Brings in cross domain learning in GAN instead of learning against noise. Model predicts novel, chemically stable ternary crystal structure	Hydride compounds
GAN	Kim *et al.*^218^	Point cloud representation consisting of cell vectors and scaled coordinates	The method generates new crystal structures for Mg–Mn–O ternary materials and evaluates their properties *via* high-throughput virtual screening	Ternary system: Mg–Mn–O
GAN	CycleGAN^240^	Image of surface showing atomic sites	Image to image generation between STM image and surface crystal structure and *vice versa*; uses discriminators between actual and generated STM image as well as surface atomic structures	Any surface
Graph attention transformer + GAN	EquiformerV2 (ref. 219)	Equivariant graphs	The model uses self-supervised learning of masked crystal structures representations, which are then fine-tuned for downstream tasks such as stability classification and regression	Inorganic crystals
GAN	GAN-DDLSF^220^	Continuity vector matrix consisting of cell vectors and atomic coordinates	Optimize the latent space *via* data-driven fusion to mitigate mode collapse of GANs	Gallium nitride Ga_*x*_N_*y*_ compositions
VAE	FTCP (Fourier transformed crystal Properties)^221^	Matrix with real and reciprocal-space features with element property matrix	Using a VAE with property-structured latent space (both input and latent space have combined lattice and property data), demonstrates generation of novel inorganic crystals at user-defined formation energy, bandgap, TE power factor	Inorganic crystals
Diffusion-VAE	CDVAE^215,223^ (Fig. 8B)	Direct coordinate representation with an equivariant graph network (node = atom, edges = bonds)	Trains the diffusion-VAE (GNN encoder, diffusion based decoder) on a database of 2D crystals, then generates new 2D materials	2D materials
Diffusion-VAE	Con-CDVAE^224^	Equivariant graph network	Extends CDVAE framework to allow target properties (band gap, formation energy, *etc.*). Implements a two-step training (first building property-aware latent space, then generating structures)	Inorganic crystals
Diffusion-VAE	Cond-CDVAE^216^ (Fig. 8C)	Matrix with atomic species, coordinates and lattice vectors	Trained on 670 000 materials from Calypso dataset; enables user-defined composition and pressure to generate physically plausible, stable crystal candidates	Inorganic crystals
VAE	WyCryst^222^	Wyckoff position-based representation	Enforces space group symmetry *via* Wyckoff positions. Combines a VAE with DFT refinement to generate stable, symmetry-compliant structures	Inorganic crystals
Diffusion using a three-channel matrix representation	Supercon-Diffusion^217^ (Fig. 8E)	A three-channel matrix that encodes the stoichiometry (integer, first decimal, second decimal) of superconductors	The method accurately learns doping characteristics, achieving high doping effectiveness and electrical neutrality, and proposes 200 new potential high Tc superconductors	Doped high Tc superconductors (cuprates, iron-based, *etc.*)
Guided diffusion model	GaUDI^241^	Molecules are represented using a graph-of-rings (GOR) representation that captures ring connectivity and geometry	Combines an equivariant graph neural network for property prediction with a diffusion denoising process	Organic molecules
Diffusion model with Riemannian manifold	CrystalGRW^225^	EquiformerV2: equivariant GNN	Framework with geodesic random walks to denoise random noise into crystal structures; preserves crystallographic symmetry and enables conditional control (*e.g.*, specifying point groups) to generate novel crystals with desired properties	Inorganic crystals
Diffusion	MatterGen^55^ (Fig. 8D)	Tuples of atomic species, coordinates and lattice vectors	The forward diffusion process corrupts an input structure. An equivariant score network does reverse denoising process with an adapter module that guides to target chemistry, symmetry, and scalar properties (*e.g.*, band gap, bulk modulus)	Inorganic crystals
Diffusion	SymmCD^226^	Space group, lattice parameters, asymmetric unit coordinates (fractional), site symmetries	A diffusion model that explicitly encodes crystallographic symmetry *via* asymmetric units and site symmetries, enabling diverse yet valid crystal generation	Inorganic crystals
Diffusion	WyckoffDiff^227^	Protostructures: String representations with space group and Wyckoff positions	A discrete diffusion model that generates symmetry-constrained protostructures using Wyckoff positions, enabling fast generation of thermodynamically stable crystals	Inorganic crystals
Transformer	BLMM^231^	Text representation for stoichiometry	A blank-filling LLM trained on ‘material grammars’	Inorganic crystals
Transformer + UMLIP	Material transformer generator (MTG)^232^	BLMM^231^	Two transformer architectures simultaneously generate material compositions which are then relaxed with M3GNET UMLIP to predict structures	2D materials
GPT2	ATOMGPT^233^	ALIGNN graph	Bidirectional prediction of structure-to-property and property-to-structure	Superconducting materials
RNN	Xiao *et al.*^52^	SLICES: String-based crystal representation ensuring symmetry invariance and invertibility	Uses a transfer learning framework to train RNN on materials project dataset for learning SLICES representations. A transfer learned RNN then predicts SLICES for novel semiconductors	Direct gap crystalline semiconductors
Transformer	Wyformer^58^	Wyckoff representation consisting of point group notations for each atom	A permutation-invariant transformer generates symmetry aware representations of novel materials for each space group	Inorganic crystals
Transformer	Matra-Genoa^57^	Sequence containing composition, lattice, Wyckoff position tokens and atomic coordinates	An autoregressive transformer that conditions generation on target properties (*e.g.*, energy above the convex hull) to produce stable crystal structures	Inorganic crystals
Transformer	CrystalFormer^242^	Sequence that integrates space group numbers, Wyckoff letters, chemical species, fractional coordinates, and lattice parameters	An autoregressive transformer that exploits space group symmetry to reduce the degrees of freedom in crystal generation. It shows improved performance for symmetric structure initialization, element substitution, and property-guided design	Inorganic crystals
Wasserstein GAN + VAE	WGAN-VAE^238^	Voxel-based representation capturing both atomic positions and lattice parameters using VAE	WGAN generates thermodynamically stable structures whereas VAE retains chemical validity	Vanadium oxide V_*x*_O_*y*_ compositions
VAE + GAN + diffusion	VGD-CG^237^	One hot encoding of composition and properties like band gap	A generator consisting of VAE, GAN and diffusion model (VGD-CG) generates compositions of target materials. A template-based structure prediction algorithm then predicts the crystal structures	Inorganic crystals
Diffusion + autoregressive token prediction	UniGenX^59^	Text sequence consisting of chemical formula, lattice vectors and atomic coordinates	The diffusion model improves the precision of prediction, whereas attention based autoregression excels in predicting sequences	Inorganic crystals, organic compounds

#### 4.2.1 GAN-based approaches for materials design

**Fig. 8:**
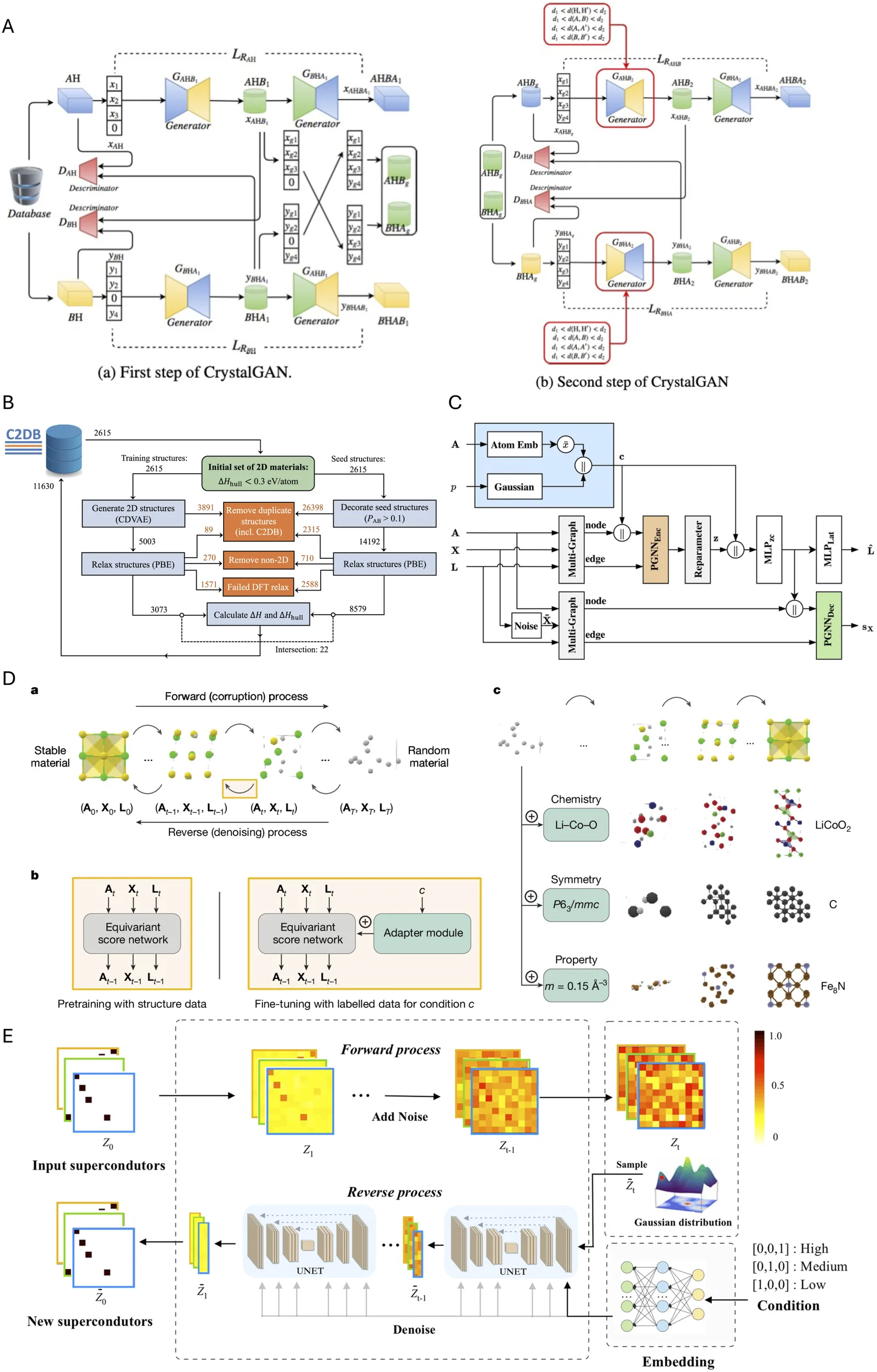
Generative inverse deep learning methods. (A) CrystalGAN architecture, adapted from ref. 214, *arXiv*, preprint, 2018. (B) Workflow for generating 2D material candidates using the CDVAE generative model, reproduced from ref. 215 with permission from Springer Nature, *npj Comput. Mater.*, 2022. (C) Architecture of Cond-CDVAE, adapted from ref. 216, *arXiv*, preprint, 2024. (D) Inorganic materials design with MatterGen, reproduced from ref. 55 with permission from Springer Nature, *Nature*, 2025. (E) Supercon-Diffusion architecture, adapted from ref. 217 with permission from Wiley, *InfoMat*, 2024.

GANs were used early in materials discovery due to their unique generator-discriminator dynamic. CrystalGAN^214^ (see Fig. 8A for architecture) introduced learning across different chemical domains instead of starting from random noise, allowing the prediction of stable crystal structures. This method has been useful for studying complex material systems like metal hydrides. Kim *et al.*^218^ extended GANs to ternary materials, using a model with three components: a generator, a critic, and a classifier. The generator uses a random Gaussian noise vector and a one-hot encoded composition vector to create 2D point cloud representations of crystal structures, guided by target compositions, while the critic measures realism *via* the Wasserstein distance. The classifier ensures the generated structures match the intended composition, with its loss back-propagated to the generator, forming a complete pipeline that generates and validates new crystal structures through high-throughput screening. Recent architectural innovations include EquiformerV2,^219^ which incorporates self-supervised learning of masked crystal structure representations through equivariant graph attention transformers, enabling more accurate assessment of crystal stability. Another advancement, GAN-DDLSF,^220^ addresses the persistent challenge of mode collapse in GANs – where the generator produces similar outputs, limiting the diversity and usefulness of generated crystal structures for materials design – by using data-driven latent space fusion (DDLSF) to enhance the variety and quality of generated materials.

#### 4.2.2 VAE-based approaches for inverse materials design

Variational autoencoders provide a powerful framework for learning compressed latent representations of material structures, while enabling generation of new candidates. The FTCP (Fourier Transformed Crystal Properties) approach by Ren *et al.*^221^ exemplifies this potential by incorporating structural and property information in a unified latent space. Their VAE architecture encodes materials using matrices combining real and reciprocal-space features with elemental properties, creating a structured latent space that enables targeted generation of inorganic crystals with user-defined properties (*e.g.*, formation energy, bandgap, *etc*). VAEs have also been used for generating lattice structure while respecting space group symmetries with help of Wyckoff position based representation of structures.^222^ For 2D materials specifically, the CDVAE (Crystal Diffusion Variational Autoencoder) framework^215,223^ represents a significant advancement. This architecture combines an equivariant GNN encoder with a diffusion-based decoder, trained directly on 2D crystal structures. By learning the relationship between atomic coordinates and material properties, CDVAE enables the generation of novel 2D materials with controlled electronic characteristics. See Fig. 8B for the workflow generating 2D materials. Advanced VAE implementations have further enhanced control over generated properties. Con-CDVAE^224^ extends the CDVAE framework to allow explicit targeting of properties like band gap and formation energy through a two-stage training process – first building a property-aware latent space, then generating structures that satisfy multiple constraints simultaneously. Cond-CDVAE^216^ (see Fig. 8C for the model architecture) further improves this approach by enabling user-defined composition and pressure constraints, generating physically plausible and stable crystal candidates from large training datasets.

#### 4.2.3 Diffusion models for materials generation

Diffusion models represent the latest advancement in generative AI for materials, offering an exceptional level of control in designing new structures. MatterGen,^55^ a state-of-the-art diffusion-based framework, encodes materials universally as combinations of atomic types, lattice vectors, and fractional coordinates. As shown in Fig. 8D, its forward diffusion process gradually corrupts input structures, while an equivariant score network performs reverse denoising with adaptable modules that guide generation toward target chemistry, symmetry, and scalar properties such as band gap and bulk modulus. For superconducting materials, Supercon-Diffusion^217^ employs a specialized three-channel matrix representation (shown in Fig. 8E) to encode stoichiometry of superconductors, accurately learning doping characteristics with high effectiveness and electrical neutrality. Advanced geometric approaches like CrystalGRW^225^ implement geodesic random walks on Riemannian manifolds to denoise random noise into realistic crystal structures while preserving crystallographic symmetry. This enables conditional control over generated structures, such as specifying point groups, to generate stable novel crystals with desired properties. Multiple recent diffusion-based architectures *viz.* SymmCD^226^ and WyckoffDiff^227^ used a different approach to generate novel yet symmetry abiding structures by using Wyckoff position based representations which automatically encodes space group symmetry constraints.

#### 4.2.4 Sequence-based models for 2D materials design

Sequence-based models like RNNs and transformers have traditionally excelled with string-based representations like SMILES for organic molecules.^228,229^ Initial studies to use transformers like GPT, BERT *etc.* for inorganic material design have shown promise as the models predicted chemically valid, charge-neutral materials, although only being able to predict compositions due to inherent limitations.^230^ One of the earliest studies for designing 2D crystals with transformer architecture was reported by Dong *et al.* wherein they first use a ‘materials grammars' aware blank-filling language model BLMM^231^ to generate target material composition and then use two ML modules trained on structure-stoichiometry data to predict most probable crystal structure for the target material.^232^ AtomGPT, a study based on GPT2 is capable of bidirectional structure-to-property and property-to-structure prediction.^233^ The model uses ALIGNN structure representation and text descriptions of structures from ChemNLP and Alpaca for inverse design of superconducting materials. Another study CrystalLLM,^234^ an autoregressive LLM, trained on a vast dataset of structures in CIF format predicts plausible crystal structure for inorganic materials. Crystal structure representation SLICES^52^ is a string-based format that maintains symmetry invariance and invertibility, enabling NLP-based transformer architectures to be applied to crystal generation. Recently, autoregressive models have started to exploit their inherent ability to interpret and generate sequences by integrating Wyckoff position based textual representation. Multiple transformer architectures using Wyckoff sequences were reported in the present year.^57–59^ A recurrent NN based transfer learning framework has been used for inverse design of narrow-gap semiconductors using SLICES strings as the backbone.^52^ The RNN is first trained on Materials Project and subsequently transfer learn a small dataset of semiconductors to predict SLICES strings for novel material candidates.

#### 4.2.5 Hybrid and multi-modal approaches

Hybrid and multi-modal approaches combining VAEs, GANs, transformers, diffusion models, and other methods are emerging as powerful tools for materials discovery. These hybrid frameworks typically leverage complementary strengths of individual models: VAEs excel at creating efficient latent representations, GANs enhance generated sample quality, diffusion models provide improved precision, and transformers offer robust sequence generation capabilities.

For example, CDVAE model^215,223^ and its extensions integrate a diffusion-based decoder within a VAE architecture, as discussed in Section 4.2.2. Recent studies have explored combining VAEs with Deep Kernel Learning (DKL), where the VAE's generative capabilities are aligned with target properties *via* Gaussian process regression in the latent space, facilitating generation of materials with specific properties.^235,236^

Further hybridization involving GANs, VAEs, and diffusion models has been demonstrated for the inverse design of target material compositions.^237^ Microsoft researchers have successfully combined the sequence modeling strengths of autoregressive transformers with the precision offered by diffusion models, enabling the generation of diverse organic and inorganic materials using Wyckoff representations for crystalline structures.^59^ Another notable approach involves integrating a Wasserstein GAN and VAE, with the GAN generating candidate structures and the VAE ensuring chemical validity, as demonstrated in the accelerated discovery of stable vanadium oxide compositions.^238^

Moreover, integration of generative large language models (LLMs) with high-throughput experimental data has been applied to the inverse design of doped perovskites. Here, a fine-tuned LLM trained on ferroelectric domain-specific knowledge constructs knowledge graphs linking structural phases, synthesis conditions, and desired properties, significantly enhancing targeted material discovery.^239^ These hybrid methodologies illustrate the growing potential of multi-modal approaches to overcome individual model limitations and accelerate advanced materials discovery.

### 4.3 Multi-objective optimization

MOO provides a powerful framework for designing 2D materials by balancing conflicting properties, yielding a Pareto front of optimal trade-off solutions. While early MOO efforts laid foundational groundwork, such as using genetic algorithms to optimize bandgap and mass in 2D phononic crystals,^243^ or differential evolution for inverse design,^244^ the integration of MOO with DL has significantly enhanced its capability, particularly for navigating through the complex design space of 2D materials.

Recent advancements highlight the synergy between MOO and DL, enabling significant progress in material design and discovery. For instance, Krishnamoorthy A. *et al.* employed NSGA-III to parameterize interatomic potentials for MoSe_2_, simultaneously optimizing structural and thermal properties while quantifying thermal conductivity uncertainty.^245^ Similarly, Varasteanu & Kusko combined NSGA-II with the transfer matrix method to enhance the sensitivity and reflectivity of 2D materials, modified surface plasmon resonance sensors, achieving configurations like Ag–BaTiO_3_–graphene/WS_2_.^246^ Zhang *et al.* utilized a multi-objective generic algorithm to parameterize potentials for MoSe_2_, improving transferability for large deformation and fracture simulations.^247^ Additionally, Jablonka *et al.* introduced a bias-free active learning algorithm using Pareto dominance to reconstruct the Pareto front for polymer design, in principle adaptable to 2D systems, drastically reducing evaluation needs.^248^

Contemporary developments further emphasize the power of coupling DL with MOO. Roy *et al.* employed multi-objective Bayesian optimization (MOBO) with active learning to identify Pareto-optimal 2D material compositions, reducing the search space by up to 36%.^249^ Chen *et al.* optimized the optical properties of liquid-phase exfoliated MoS_2_ using a genetic algorithm-coupled artificial neural network, precisely tuning absorbance and bandgap.^250^

Although much recent work emphasizes optimizing structural, thermal, or optical properties, the potential of MOO for electronic structure design is also very promising. By simultaneously considering multiple electronic parameters – such as effective mass, bandgap size and band alignment – MOO can efficiently address inherent trade-offs critical to electronic applications. For example, incorporating MOO with a compound loss function into DL could accelerate the discovery of 2D materials with tailored electronic properties for next-generation electronics, where trade-offs between different electronic characteristics are often necessary. A recent study demonstrated this capability by leveraging Wyckoff position augmentation and transfer learning to optimize for targeted space group characteristics, bandgap, and formation energies, ultimately predicting several stable structures.^251^

As 2D material datasets continue to grow and computational resources expand, we anticipate that MOO coupled with DL will become increasingly central to electronic structure design, enabling researchers to navigate complex property spaces and identify optimal candidates for specific technological applications.

## 5 Opportunities and future directions

The exciting advances in deep learning for 2D materials research offer a wealth of opportunities. Yet, significant challenges remain. DL models have already shown great promise, but their ability to generalize beyond specific datasets still needs improvement.^154,252–254^ This is especially important for 2D materials, known for their diverse behavior and unique properties arising from intricate interactions between chemistry, structure, and physics. Improving model architectures and ensuring high-quality data will be key to unlocking broader generalization and driving further breakthroughs in this vibrant field.

A key area for future advancement is improving the interpretability of deep learning models. As these models become increasingly complex, understanding their decision-making processes is essential for scientific validation and building trust. Promising approaches include physics-aware DL and explainable artificial intelligence (XAI), which integrate fundamental physical and chemical principles directly into model architectures.^14,205,255^ Such strategies not only enhance predictive accuracy but also offer valuable scientific insights, enabling researchers to better understand underlying mechanisms and develop hypotheses guided by model outputs.

Another promising direction involves improving data accessibility, comprehensiveness, and standardization. Major databases like Materials Project, 2DMatpedia, and C2DB have been instrumental in accelerating materials discovery; however, there remain significant opportunities to expand their scope, particularly by incorporating more comprehensive electronic structure data such as wavefunctions. Enriching these databases with detailed datasets would enable advanced computational analyses, especially for exploring novel quantum and topological phenomena. We suggest establishing clear and standardized reporting protocols, complemented by structured ontological frameworks (*e.g.*, MatOnto,^256^ MatOWL,^257^ and others^258,259^) specifically designed for 2D materials. Such frameworks would significantly enhance data usability, making it easier for researchers to find, interpret, and apply relevant information. Furthermore, embracing semantic web technologies like JSON-LD^260^ or Splink^261^ could greatly improve data interoperability. This would facilitate automated reasoning and seamless integration across diverse datasets, enabling more reliable benchmarking and validation efforts. By comprehensively standardizing structural parameters, electronic properties, synthesis metadata, computational methodologies, uncertainty quantification, and validation procedures, the research community can substantially amplify the impact and efficiency of DL-driven autonomous experimentation in materials science.

Another direction of improvement is ensuring DL predictions are consistent with experiments. Initial data-based DL models used to be purely trained on data from high-throughput first-principles calculations. Their accuracy is therefore directly related to the accuracy of the DFT data. However, later on, with the rise of physics-aware DL models like equivariant GNNs and message-passing architectures, the DL models try to predict with the awareness of the physical structure, thus being more accurate over out-of-training regime. Furthermore, accurate datasets, which closely mimic experiments, *e.g.* coupled cluster methods, GW, hybrid methods, and dynamic mean field theory, are also being progressively incorporated in training to reach experimental level accuracy. However, large experimental datasets with electronic properties are still missing, preventing training DL models for realistic electronic structures. Some experimental datasets on band-gaps are available, *e.g.* Matbench_expt_gap,^75^ and the dataset created by Google DeepMind.^262^ Another way to reach experiment-level accuracy is through the use of self-driving labs in which AI plans experiments, executes them *via* robotics, analyzes results, and then plans new experiments to train DL models in real time. We discuss this topic further below.

The emergence of foundation models – large-scale pretrained AI systems capable of performing multiple tasks – offers exciting new opportunities for 2D materials discovery. Although this field is still developing, foundation models have immense potential to address some of the most challenging issues facing materials science today. These challenges include diverse representations of materials data, the need to handle physics across multiple length scales, varied computational approaches, and complex interdependencies between different material properties.^263^ AFLOW-ML^48,264^ offers early capabilities for property prediction across material classes. The NIST-JARVIS framework has evolved to support numerous materials informatics tools and property prediction modules. The MACE architecture^265^ demonstrates foundation-like capabilities for molecular dynamics simulations, phonon spectra prediction, and battery modeling. The MLMD platform^82^ provides an active learning system for multiple property predictions without requiring specialized coding. Recently developed transformer-based models like MatterGPT or AtomGPT have already demonstrated encouraging progress, showing their capability to generalize across a wide range of materials properties and tasks.^233,266–268^ Additionally, innovative methodologies such as symbolic regression present intriguing possibilities for creating transparent, interpretable models.^269,270^ These approaches not only help researchers build clearer connections between theory and experiment but also foster deeper scientific insights into the underlying physical phenomena.^271–273^

Transformer-based large language models have also shaped the materials discovery landscape in last few years using their incredible ability to utilize vast cross-domain knowledge. Text only transformer models like,^71^ and bigger models like Llama^274^ are enabling reliable extraction of synthesis conditions, substrate choices, and performance metrics for graphene derivatives, TMDs, MXenes, and other van der Waals systems. These information then can be fed into dataset creation, inverse-design loops and lab automation. Some big examples of LLMs are Concensus, Scite,^275^ Elicit^276^ which are trained on large amount of scientific literature and materials databases. Multimodal foundation models extend this by fusing text with images *e.g.* surface images from TEM/AFM, diffraction, and spectroscopy (Raman/PL/ARPES) to learn both atomic structure and electronic signatures for 2D materials. Gemini, GPT-5, NotebookLM are examples of such multi-modal models. Together, these text-based and multi-modal models are helping to build structured knowledge-base to revolutionize 2D materials design.

Combining advanced AI into fully autonomous materials discovery holds immense potential to revolutionize innovation in 2D materials.^5,6^ A self-driven laboratory with cutting-edge AI models could propose entirely new classes of 2D materials, carefully tailored for specific applications. Once a promising candidate is identified, robotic systems will carry out precise synthesis and characterization experiments automatically. Every piece of data collected – whether successful or not – feeds back into AI, adjusting their models and improving the quality of future proposals. This iterative, closed-loop process is already seen in early experimental systems which demonstrates the feasibility of these autonomous workflows. These pioneering examples showcase how continuous interaction between AI-driven prediction and robotic experimentation can rapidly refine materials design and discovery. Looking ahead, fully integrated autonomous labs could dramatically accelerate the identification and development of new materials, going far beyond human speed and efficiency. Ultimately, this integration promises to transform materials science – not merely accelerating current methods, but enabling entirely new approaches, materials, and scientific breakthroughs previously unimaginable.^36,277^

Finally, quantum computing represents a fascinating emerging frontier, poised to greatly influence the future of materials discovery. Current classical computational methods often face major bottlenecks – especially when simulating strongly correlated electronic systems common in many 2D materials.^278^ Hybrid quantum-classical algorithms^279^ and quantum embeddings methods^280^ offer promising new ways to tackle these longstanding challenges. As quantum technologies mature, combining them effectively with existing AI approaches may open doors to breakthroughs that were previously unattainable. This powerful integration could redefine our understanding of materials and accelerate the discovery of novel phenomena.

Together, these opportunities paint a compelling vision of a future where discovering, characterizing, and optimizing new 2D materials is dramatically faster and more effective. Powered by advanced agentic AI systems, autonomous experimentation platforms, and breakthroughs in quantum computing, researchers will rapidly unlock practical innovations across diverse technological fields – from electronics and energy to quantum technologies and healthcare.

Despite their promise, current DL approaches face important limitations. Most models lack robust uncertainty quantification, making it difficult to assess predictive reliability, particularly when extrapolating beyond the training data. They are also prone to biases inherited from limited or imbalanced datasets, and their interpretability remains a bottleneck for extracting physical insight rather than just numerical predictions. Additionally, the growing computational cost and associated carbon footprint of training large-scale models raise ethical and sustainability concerns in the long term. Addressing these challenges, through improved uncertainty-aware architectures, physics-informed learning, interpretable model design, and more energy-efficient training protocols, will be essential for ensuring that DL develops as a trustworthy and responsible tool for materials discovery.

## 6 Conclusions

This review highlights the transformative impact of deep learning in studying and predicting electronic properties of 2D materials. DL models have rapidly evolved into powerful tools. They not only accelerate computational predictions but also unveil subtle electronic phenomena often missed by traditional methods.

Combining DL with physics-aware models and autonomous experimentation represents a significant advancement. This integrated approach offers deeper scientific insights, facilitating the discovery and development of novel materials tailored for specific applications. Furthermore, emerging technologies such as hybrid quantum-classical computing expand the potential of DL and related AI approaches. Such methods are particularly valuable for simulating complex electronic interactions in strongly correlated materials – an area traditionally challenging for classical computation alone.

Looking forward, dedicated efforts toward standardizing data, innovating new methodologies, and developing autonomous experimentation platforms will be critical. These advancements will ensure DL evolves from merely a computational aid into a fundamental aspect of research strategies, greatly enriching our scientific understanding and practical utilization of 2D materials.

## Author contributions

Conceptualization: AM. writing – original Draft: AM, AB, XW, HKP, YW. writing – review & editing: AM, AB, XW, YW, QY. visualization: AB, XW, YW. supervision: AM, QY. project administration: AM. funding acquisition: AM, QY.

## Conflicts of interest

The authors have no conflict of interest to declare.

## Data Availability

No primary research results, no software or code have been developed, and no new data were generated or analyzed as part of this review.
